# Haptic virtual surgery simulation system under field programmable analogue array-based hybrid control

**DOI:** 10.1038/s41598-022-16655-9

**Published:** 2022-07-20

**Authors:** Sun Ru, Ting Yang, Liang Zhang, Lin Wang, Yili Fu, Mahdi Tavakoli

**Affiliations:** 1 School of Medical Information and Engineering, Xuzhou Medical University, Xuzhou, 221004 Jiangsu China; 2 Key Laboratory of Robotics and Systems, Harbin Institute of Technology, Harbin, 150080 Heilongjiang China; 3 Department of Electrical and Computer Engineering, University of Alberta, Edmonton, T6G2V4 Canada; 4 School of Information and Control Engineering, China University of Mining and Technology, Xuzhou, 221116 China; 5 Guangdong Key Laboratory of Intelligent Information Processing, Shenzhen Key Laboratory of Media Security, Shenzhen, 518060 China

**Keywords:** Biomedical engineering, Electrical and electronic engineering

## Abstract

In this paper, a bilateral haptic virtual surgery simulation system under a hybrid controller was studied. An analogue controller realized by a field programmable analogue array (FPAA) was paralleled in the operator robot side, which reduced the impact of controller discretisation on the system. A system stability conditions under hybrid control with multiple-operators were deduced. The stability analysis indicates that the addition of analogue derivative term widens the range of haptic controls gains that satisfy the multiple-users’ stability conditions. Finally, the human’s performance of a stiffness discrimination task was studied in an independently developed minimally invasive surgical (MIS) platform. The experiment results show that, human operators under the hybrid controller achieve the highest task success rates.

## Introduction

To save the training cost and accelerate the operator’s familiarity with the functions of the tele-operation system, the studies on the supporting haptic virtual tele-operation simulation training system is essential. The haptic virtual surgery simulation system is composed of the operator, virtual environment, and haptic human-computer interaction device. The haptic human-computer interaction device connects the former two parts and transmits the virtual dynamic perception to the operator. The main applications include haptic human-computer interaction systems of fabrics, virtual surgery simulation systems, virtual games, and remote rehabilitation systems^1–4^.

Only when the bilateral virtual training system controller is designed to ensure sufficient system transparency, the operator has perception and performance similar to those in direct execution. On the premise of ensuring system stability, this paper designed a new bilateral virtual system controller to increase the task success rate of the tele-operation system and its training system. In 2015, Kim et al. studied the stability of the multiple-degree-of-freedom haptic virtual simulation system using the force boundary limiting method^5^. This method used three sufficient stability conditions to limit contact vibrations, ensuring the passivity and stability of the human-computer interaction system. In 2019, Yang Hongjiu et al. proposed a tracking control strategy with an extended state observer, a time-delay part observer and a continuous terminal sliding mode control^6^. And in 2020, Amir Zakerimanesh et al. developed their nP+D like controllers for nonlinear bilateral teleoperation with time-vary delays which exhibits better transient error in position convergence^7^.

### Effect of controller discretisation on tele-operation system transparency and its solution

Because the sampling process required for simulating the virtual environment compromises the system stability conditions, the first thing to consider in a bilateral haptic virtual surgery simulation system is its overall stability. The research on energy leakage caused by a sample and hold showed that the zero-order hold (ZOH) could lead to the delay of half sampling period and the accumulation of system energy^8,9^.Therefore, in a bilateral tele-operation system with a discretised controller, the effect of sampling on system stability and transparency cannot be ignored^10^.The studies present on bilateral control algorithms have focused on the common non-ideal situations such as friction, noise, control signal overflow, un-modelled dynamics, communication network delay, and active or passive systems. However, a few studies have reported the effect of discretisation on system performance, especially transparency. Simultaneously optimising the system stability and operability of the bilateral tele-operation control system is challenging in practical applications.

For bilateral virtual training and simulation systems, scientists have used virtual coupling impedance between the haptic device and virtual object to form a virtual control system^11,12^. Because the operator can only contact the human-computer interaction device, the instability of the system may lead to wrong perception or task failure. Therefore, ensuring the stability of the control system in a human-computer interaction device is also essential. Minskky et al. reported the compensation effect between the simulation sampling rate, virtual wall stiffness, and viscoelasticity of a human-computer interaction device^13^. Colgate and Schenkel et al. studied the passivity of a haptic virtual surgery simulation system with a movable operator and simple virtual wall objects and tool equipment and reported the conditions for system passivity^14^. The aforementioned studies showed that the root cause of system instability is the sampling process of the control system. The longer the sampling period, the more difficult to ensure system stability and the lower the coupling impedance. In other words, when the sampling period is large, the operator might feel a soft touch while encountering stiff virtual objects, resulting in obvious perception errors and unsatisfactory system transparency.

To minimise the sampling period in complex computing and simulating environments, one strategy was to use a block computing structure. For example, multi-threaded processing simulated the overall environment in a long sampling period but used a small sampling period near deformed parts or the virtual human-computer interaction device^15^. However, this method still failed to ensure that the local simulation met the task requirements. Another common strategy was to use a fast sampling processor^16–19^. Hannaford and Lawrence et al. addressed the contradiction between transparency and other properties such as the stability of tele-operation system^20^. Hace et al. used a small sampling period with the field programmable gate array (FPGA), which could optimise system performance to a certain extent^21^. However, this method was more expensive than the commonly used personal computer and it could not solve the problem of gain limit.

A more affordable strategy was to use analogue components to improve the controller of the telecontrol robot. Since an analogue system does not need sampling signals, the complete stability condition of the system does not limit the maximum analogue control gain^22^. In other words, unlike in a digital control system, the transparency and stability in an analogue system were not restrictive to one another. Therefore, this paper hopes to add analogue control in the tele-operation system and fundamentally eliminate the limit caused by the sampling period.

### The field programmable analogue gate array (FPAA)

The field programmable analogue array (FPAA) used in this study greatly simplified the design process of the analogue controller and reduced the debugging time for the analogue circuit. Unlike common digital programmable circuits (e.g., FPGA or complex programmable logic device, CPLD), the analogue programmable circuit is newly developed in the recent two decades. Various commercial FPAA circuits are available in the market, and the number of researchers joining in the research and development of FPAA is increasing^23–25^.

Plenty of studies used FPAA chips developed by Anadigm Company and focused on the applications in control and measurement systems, audio signal processing, and biomedical signal processing^26,27^. With its upper-level AnadigmDesigner2 development software, designers can use the functional configurable analogue module (CAM) to code complex simulation functions. This paper used two types of Anadigm dpASP devices (second-generation FPAA), AN231E04 and AN21E04, in the design of the simulation controller.

### Contributions of this paper

In practical applications, the analogue control gain is still limited by the saturation and non-linearity of the operational amplifier in the circuit, which induces an upper limit of the analogue control gain. Also, implementing complex algorithms in an analogue controller is difficult. Therefore, this paper also considers combining FPAA analogue control and digital control to remove the limitations of a single control method.

The advantages of combining analogue/digital control are analysed in the human-computer interaction environment of HVE simulation^28^. However, the stability conditions are considered only for single-user cases, rather than in a bilateral tele-operation system environment. On the other hand, among the reports on the stability analysis of bilateral tele-operation systems, few have directly studied the effect of energy leakage, i.e., the aforementioned controller discretisation^29,30^. Thus this paper used the FPAA analogue controller to solve the problem of energy leakage and added a digital controller to overcome the shortcomings of the analogue controller such as the difficulty in implementing complex algorithms.

The contributions of this paper are as follows. A haptic virtual surgery simulation control system based on the hybrid method was proposed, the stability inequalities for the virtual control system with multi-users under four delay and passivity conditions were deduced, and the gain ranges of the system with and without delay were presented.

## Methods

### Modelling of the haptic virtual surgery simulation system under hybrid control

**Figure 1:**
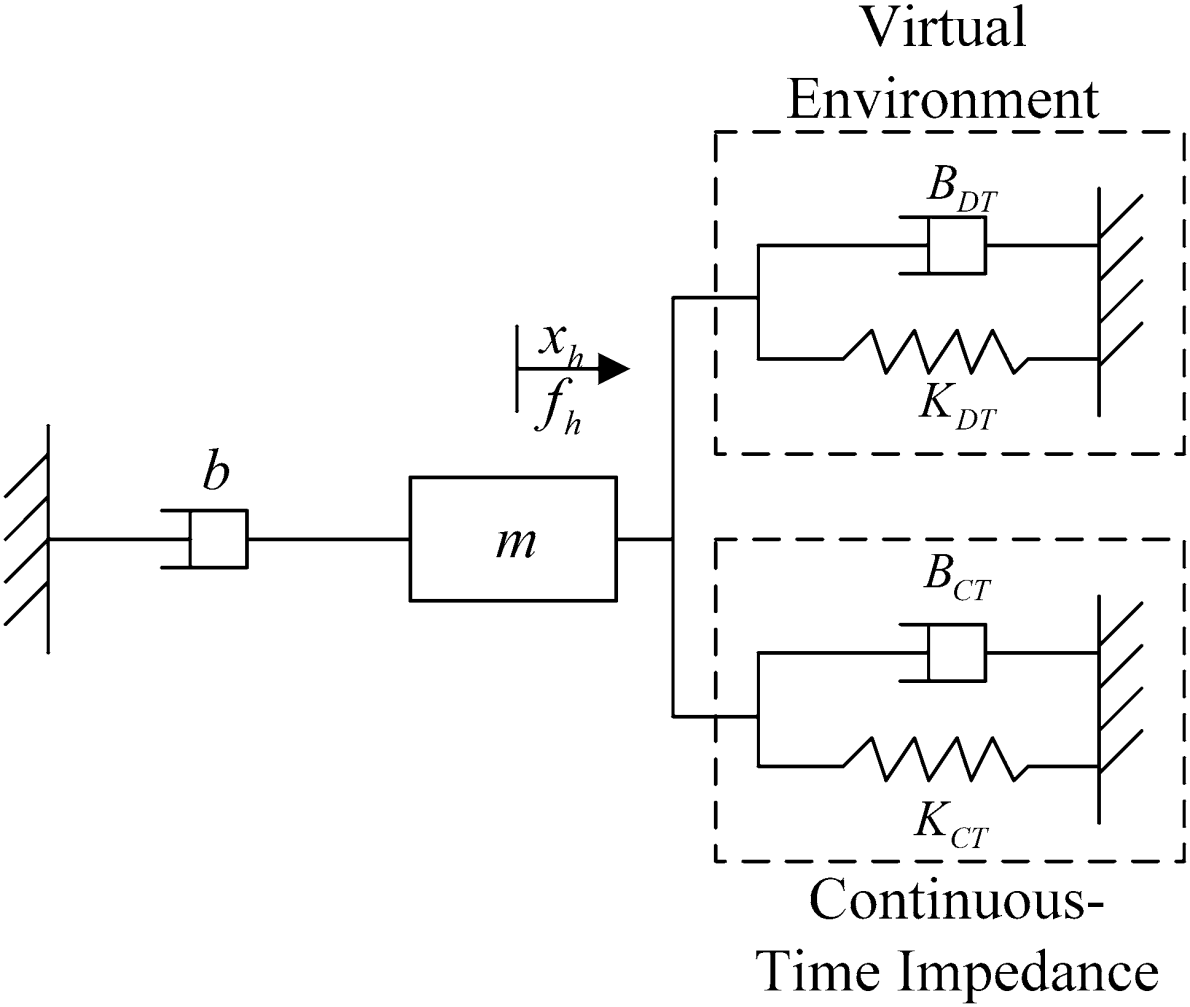
A single-user haptic virtual surgery simulation system with an analogue controller.

This section describes the establishment of a haptic virtual surgery simulation system model containing a continuous-time impedance controller, as shown in Fig. 1. $f_h$ represents the operator force and $x_h$ represents the displacement of the haptic virtual device. *m* represents the mass of the haptic virtual human-computer interaction device and *b* is the damping. $B_{DT}$ denotes the virtual damping and $K_{DT}$ denotes the virtual stiffness of the virtual environment. $B_{CT}$ represents damping for the continuous-time impedance and $K_{CT}$ represents stiffness. $H_{DT}$ denotes the transfer function of the discrete-time controller and $f_{DT}$ denotes the output force.

In Fig. 1, the operator applies a force $f_h$ to the human-computer interaction device, driving the device to move a distance $x_h$. The displacement enters the virtual environment and the continuous-time impedance. The sum of the virtual force/displacement information feedback and the simulation information feedback formed in the continuous-time impedance is fed back to the operator through the human–computer interaction device. The operator then perceives virtual information and action instructions to the virtual simulation environment. The instructions are ultimately executed in the virtual environment. The continuous-time impedance represents the analogue controller in parallel with the virtual control system, which is implemented by the FPAA in this paper.

**Figure 2:**
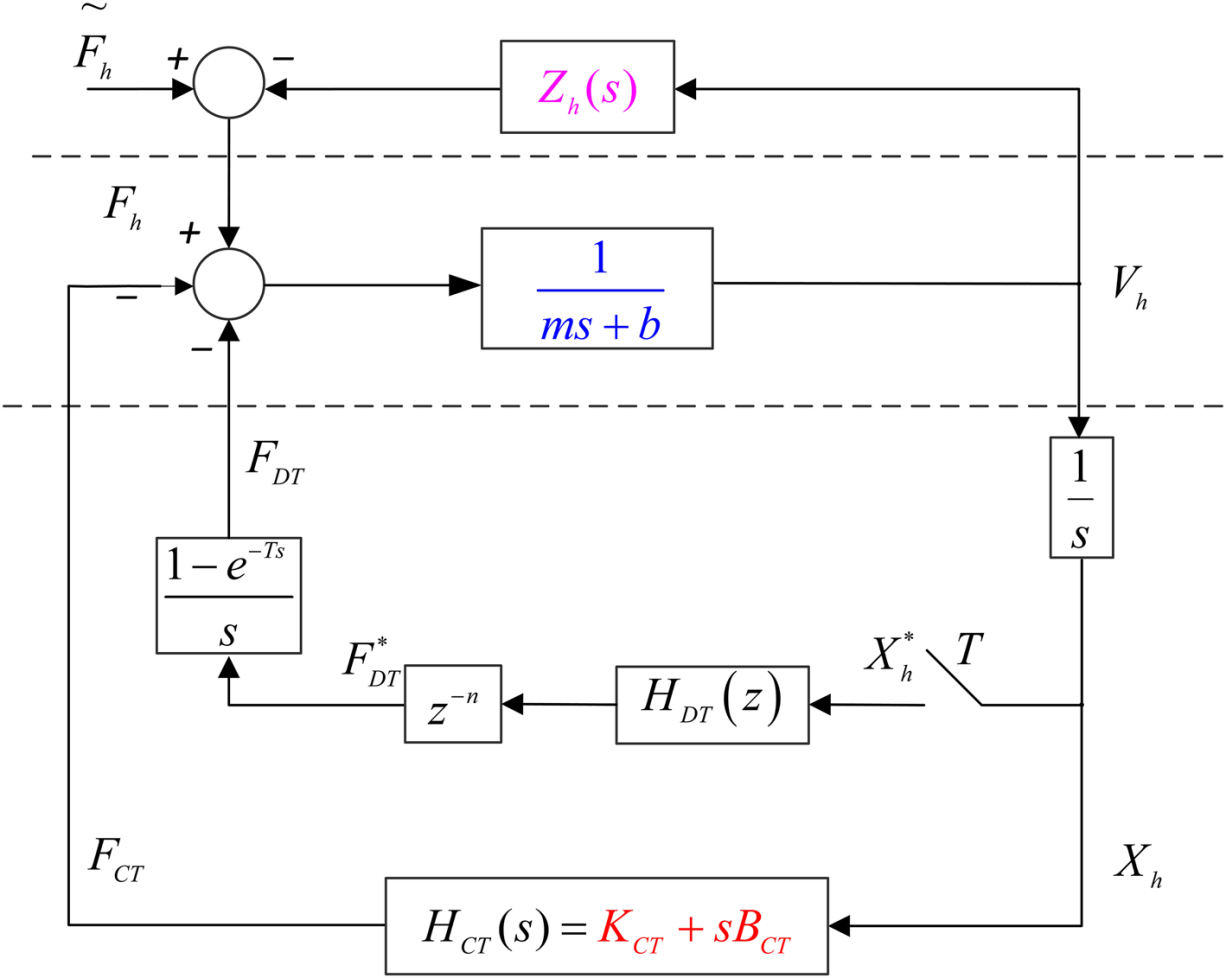
A single-user haptic virtual surgery simulation system with a hybrid controller .

After simple mathematical transformation, the single-user haptic virtual surgery simulation system under hybrid control in Fig. 1 can be represented in the form of Fig. 2. $*$ denotes signals after discretisation and *T* represents the sampling period. $F_h$ is the control force signal of the haptic virtual device in *s* domain, $F_{DT}$ is the output force signal of the digital controller, which is converted by the discrete quantity ( $F_{DT}^{*}$ ) through ZOH, and $F_{CT}$ represents the output force signal of the analogue controller. $X_{h}$ represents the displacement of the human-computer interaction device (leader-follower robot), which is converted into the discretised displacement ( $X_h^{*}$) through the sampling module. $H_{DT}(z)$ denotes the known discrete model (i.e., digital virtual coupling between the haptic virtual human-computer interaction device and the virtual wall), and $H_{CT}(s)$ represents the gain coefficient of FPAA-based analogue control:

1 $$\begin{aligned}&H_{CT}(s)=K_{CT}+sB_{CT} \end{aligned}$$

2 $$\begin{aligned}&H_{DT}(z)=K_{DT}+\frac{z-1}{Tz}B_{DT} \end{aligned}$$

$Z_h(s)$ represents an unknown operator model, which can be obtained from Fig. 2 as follows:

3 $$\begin{aligned} \tilde{F_h} - F_h = Z_h(s)\cdot sX_h \end{aligned}$$

Where *s* is the Laplace constant, $\tilde{F_h}$ denotes the external operating force. The dynamic model of the leader-follower robot in domain is as follows:

4 $$\begin{aligned} F_h - F_{CT} - F_{DT}= (m \cdot s +b)V_s \end{aligned}$$

The impedance of the haptic virtual device can be expressed as follows:

5 $$\begin{aligned} Z_h^{-1}=\frac{1}{ms+b} \end{aligned}$$

where *m* denotes the mass of the haptic virtual device and *b* denotes damping.

In the digital controller, the analogue displacement signal $X_h$ needs to be sampled first with a sampling period*T*^30^:

6 $$\begin{aligned} X^*(s) = \sum _{k=0}^{\infty } x(kT) e^{-skT} \end{aligned}$$

Equation (6) can be written as $X(z)=X^*(s) \vert _{s=1/Tlnz}$ in domain. The ZOH module uses the following transfer function to transform the output of the digital controller into the analogue signal.

7 $$\begin{aligned} G_h(s)=\frac{\left( 1 - e^{-sT} \right) }{sT} \end{aligned}$$

**Figure 3:**
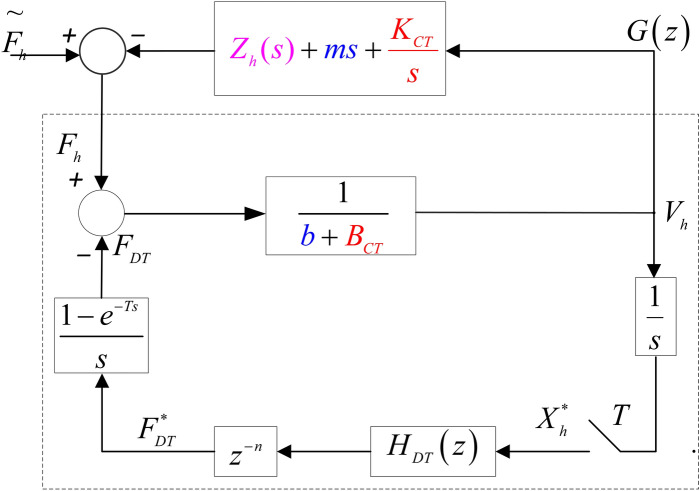
A model of single-user haptic virtual surgery simulation system under hybrid control.

The input sampling signal $H_{DT}(x)$ is output after ZOH processing with a sampling period *T*. In Fig. 2, $V_h=sX_h$ represents the motion speed of the human-computer interaction device. The velocity needs to be multiplied by 1/*s* and converted into the displacement. Fig. 3 provides a clearer form of Fig. 2 after a simple transformation as given below. First, move the mass (*m*) of the human-computer interaction device and the proportional term (multiplied by 1/*s* to become $K_{CT}/s$) of the simulation controller to the side of the operator and operation object impedance. This process is reasonable as it does not change the closed-loop transfer matrix. Then, move the proportional term ($B_{CT}$) of the analogue controller in the human-computer interaction device to the side of the leader-follower robot impedance, which is also reasonable because it does not change the closed-loop transfer matrix .

**Figure 4:**
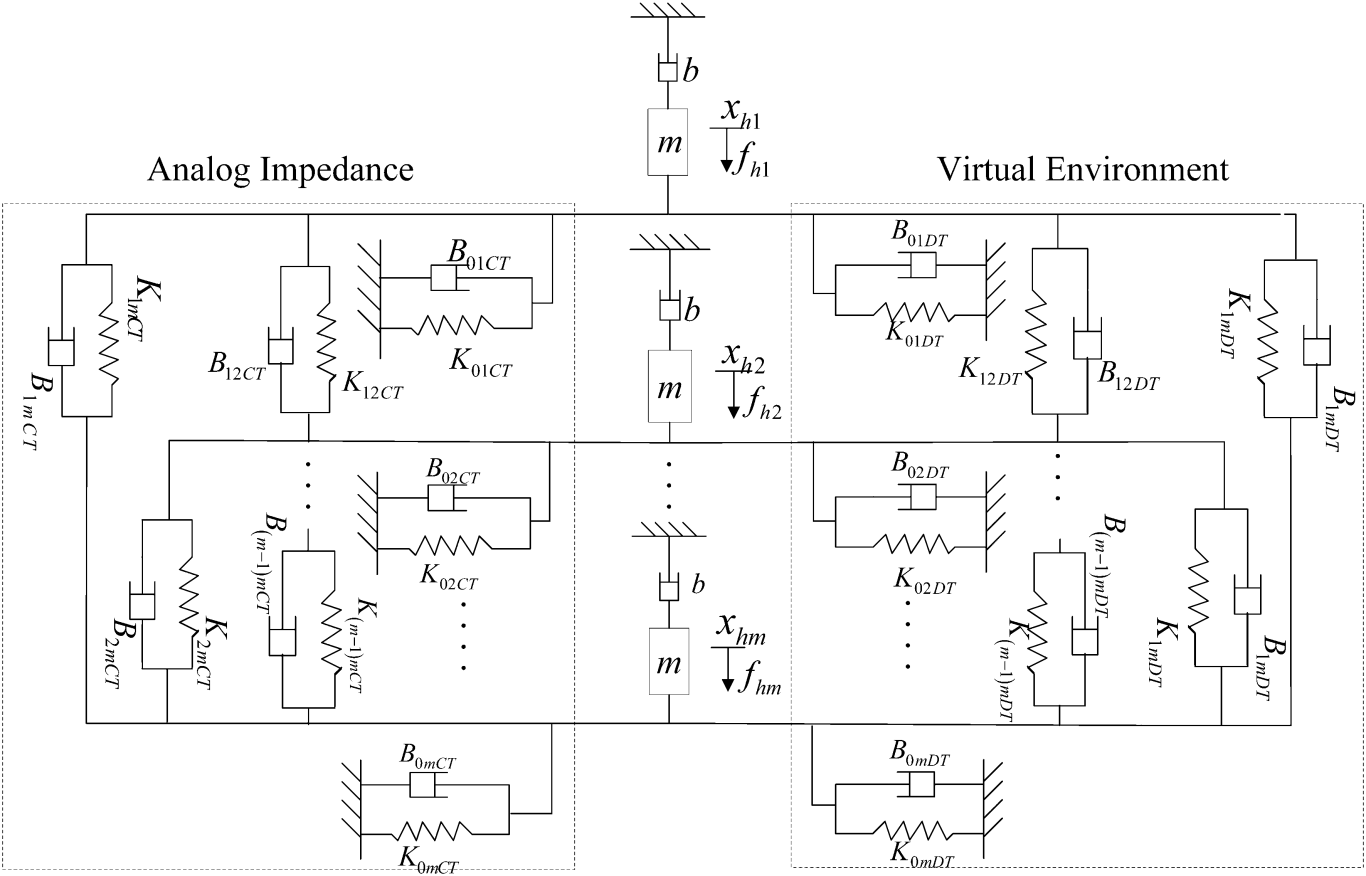
A multiple-user haptic virtual surgery simulation system with the analogue controller.

**Figure 5:**
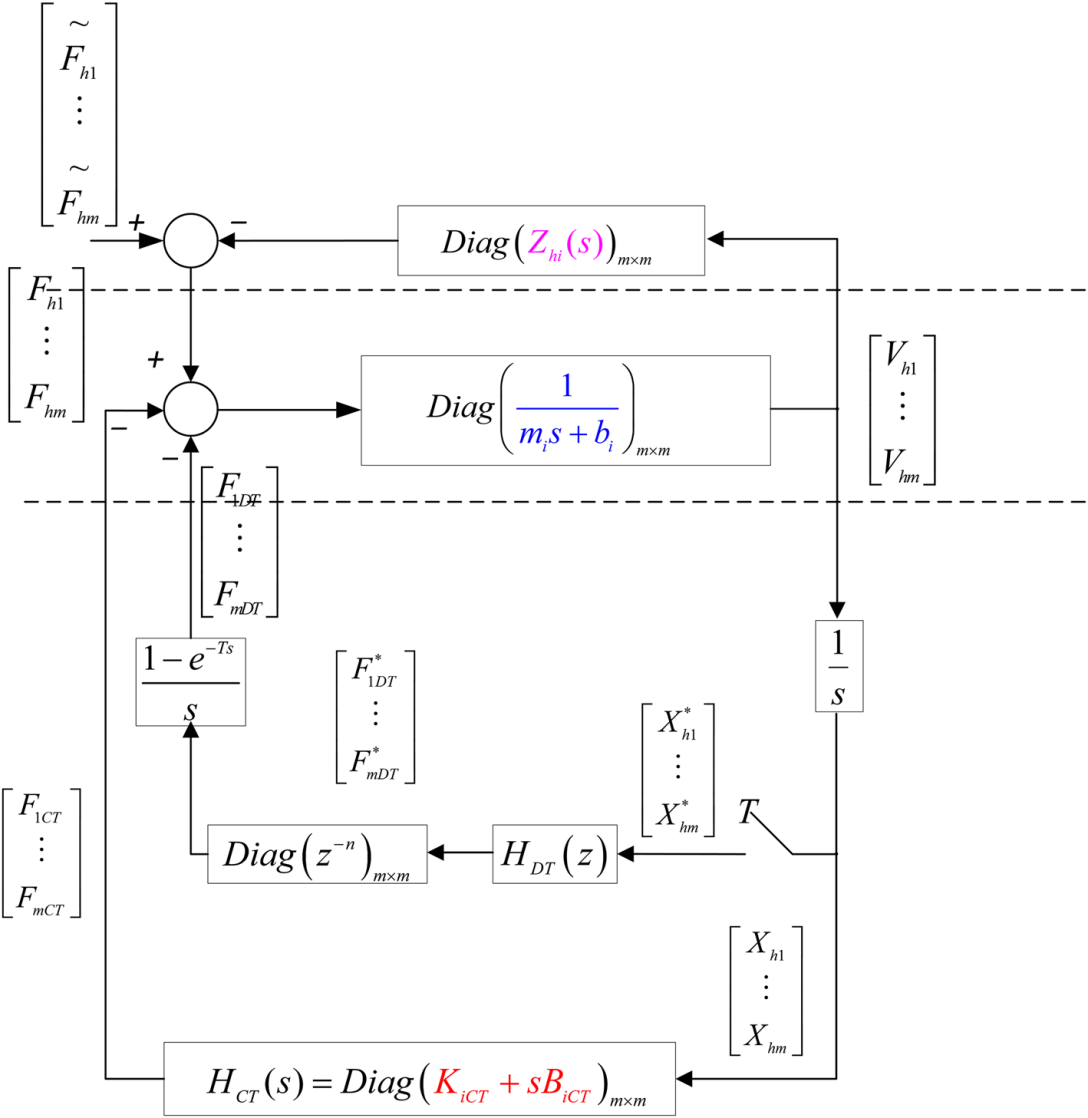
Model of a one-dimensional multiple-user haptic virtual surgery simulation system with the analogue controller.

**Figure 6:**
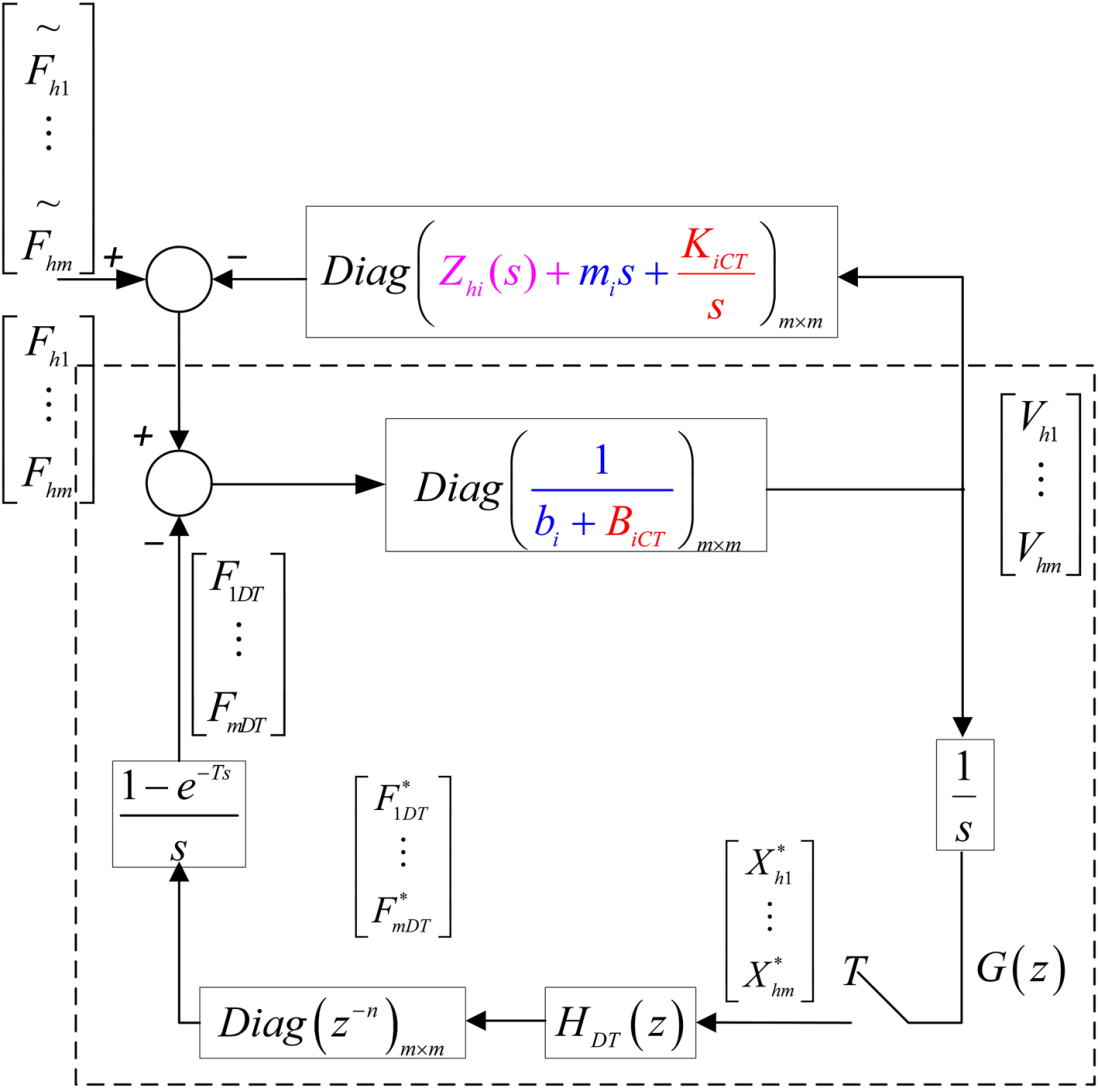
Simplified model of the one-dimensional multiple-user haptic virtual surgery simulation system with the analogue controller.

The following can be obtained according to Fig. 3:

8 $$\begin{aligned}&F_h - F_{DT}=(b+B_{CT})V_h \end{aligned}$$

9 $$\begin{aligned}&X_h=\frac{V_h}{s} \end{aligned}$$

10 $$\begin{aligned}&F^{*}_{DT}=z^{-n}H_{DT}(z)X^{*}_{h} \end{aligned}$$

Assuming that $n=t_d / T$ is an integer ($t_d$ represents communication delay), combined with Eqs. (8) and (10), it is obtained that

11 $$\begin{aligned} F_h(z)-z^{-n}H_{DT}(z)X_h(z)=(b+B_{CT})V_h(z) \end{aligned}$$

Here, $X_h(z)=Z\{ V_h / s \}$. It should be noted that $Z\{ V_h / s \} \ne Z\{ 1 / s \}V_h\{ z \}$; therefore, to obtain the transfer function from $f_h$ to $v_h$, the value of $Z\{ V_h / s \}$ should be estimated first. The estimation uses one of the three different estimation methods as described in equations (Appendix B-1, Appendix B-2, and Appendix B-3).

The impedance of the operation object is typically estimated as $H(z)=K+B(z-1) / Tz$ in $\mathrm {Z}$ domain^31–35^. Using the same estimation model, “Stability analysis of the single-user haptic virtual surgery simulation system under hybrid control” considers system stability conditions under four different scenarios, wherein the operator is passive or active and the communication delay exists or not.

After establishing the single-user simulation system model of haptic virtual surgery, the multi-user simulation system can be modelled as in Fig. 4. As each operator only affects the corresponding leader robot (haptic virtual human–computer interaction device), Fig. 4 can be transformed into Fig. 5, which can be simplified as Fig. 6.

The specific conversion method is similar to that of the single-user model; hence, the dynamic equation shown in Fig. 6 becomes as follows:

12 $$\begin{aligned} (b+B_{iCT})V_{hi}(z)=F_{hi}(z)-F_{iDT}, \quad i=1,\cdots ,m \end{aligned}$$

where $V_{hi}(z)=Z\{v_{hi}\}$, $F_{hi}=Z\{ f_{hi} \}$, and $F_{iDT}=z^{-n_i}H_{iDT}(z)X_h(z)$ represent the output force signals of the digital controller. $n_i=t_{di} / T$ is an integer, and $H_{ii}(z)=K_{0i}+\sum _{k=1,k\ne i}^{m} \left( K_{ik} + \frac{\left( B_{0i} + \sum _{k=1,k\ne i}^{m} B_{ik}(z-1) \right) }{Tz} \right)$, $H_{ij}(z)=-\left( K_{ij} + \frac{B_{ij}(z-1)}{Tz}\right) ,\quad j\ne i$. Therefore,

13 $$\begin{aligned} \begin{array}{l} {F_{iDT}}\! =\! {z^{ \!-\! {n_i}}}\left( {{K_{0i}} \!+ \!\sum \limits _{k\! =\! 1,k \ne i}^m {\left( {{K_{ik}} \!+ \!\frac{{\left( {{B_{0i}} \!+ \!\sum \limits _{k\!= \!1,k \ne i}^m {{B_{ik}}} } \right) \left( {z\!- \!1} \right) }}{{Tz}}} \right) } } \right) {X_{hi}}\left( z \right) -\! {z^{\! -\! {n_i}}}\left( {{K_{ij}}\! + \!\frac{{{B_{ij}}\left( {z\! -\! 1} \right) }}{{Tz}}} \right) {X_{hj}}\left( z \right) \end{array} \end{aligned}$$

Using forward difference estimation to replace 1/*s* in Eq. (13) and also considering Eq. (12), the relationship between the force vector $F_h$ and velocity vector $V_h$ is as follows:

14 $$\begin{aligned} F_h(z)=G^{-1}(z)V_h(z) \end{aligned}$$

where *G*(*z*) is a $m \times m$ transfer matrix of the multi-user haptic virtual surgery simulation system. Similarly, its stability is considered under four scenarios of passivity and delay.

### Stability analysis of the single-user haptic virtual surgery simulation system under hybrid control

This section discusses the complete stability of the virtual surgery training system under FPAA-based analogue and digital hybrid control. During task execution, human factors inevitably affect the passivity of the operator. Therefore, in the system framework proposed in this paper, both passive and active operators were analysed. The method used for stability analysis in this section originated from the stability condition of haptic virtual systems for single and passive operations proposed by Colgate^32^, and extended to the case of m active operators. Furthermore, the effect of communication delay on stability was considered.

#### System stability inequality in the passive, undelayed scenario

According to Fig. 3 and the three commonly used difference estimation algorithms (the forward difference method, equation (Appendix B-1); the backward difference method, equation (Appendix B-2); and the Tustin transformation method, equation (Appendix B-3), the stability of the single-user haptic virtual surgery simulation system with a hybrid controller was analysed in the passive, undelayed scenario.

When $t_d=0$, the relationship between $f_h$ and $v_h$ can be expressed as follows.

In the forward difference method,

15 $$\begin{aligned} F_h(z)\!=\!(b\!+\!B_{CT})V_h(z)\!+\!\left( K\!+\!\frac{B(z\!-\!1)}{Tz}\frac{T}{z\!-\!1}V_n(z)\right) \!=\!G_1^{\!-\!1}(z)V_h(z) \end{aligned}$$

In the backward difference method,

16 $$\begin{aligned} F_h(z)\!=\!(b\!+\!B_{CT})V_h(z)\!+\!\left( K\!+\!\frac{B(z\!-\!1)}{Tz}\frac{Tz}{z\!-\!1}V_n(z)\right) \!=\!G_2^{\!-\!1}(z)V_h(z) \end{aligned}$$

In the Tustin transformation method,

17 $$\begin{aligned} F_h(z)\!=\!(b\!+\!B_{CT})V_h(z)\!+\!\left( K\!+\!\frac{B(z\!-\!1)}{Tz}\frac{T}{2}\frac{z\!+\!1}{z\!-\!1}V_n(z)\right) =G_3^{\!-\!1}(z)V_h(z) \end{aligned}$$

If the value of *Z*[*v*/*s*] is estimated by the forward difference method, that is, $z=cos\left( (\omega - \omega _0)T \right) + jsin\left( (\omega - \omega _0)T \right)$ is substituted in Equation (15), then Condition 2 of Definition Appendix A-3 can be transformed into requiring the sum of $G_1^{-1}\left( e^{j(\omega -\omega _0)T} \right) + G_1^{-T}\left( e^{-j(\omega -\omega _0)T} \right)$ to be a positive definite matrix. According to Definition Appendix A-2, it is required to have

18 $$\begin{aligned} b+B_{CT} > \frac{K_{DT}T}{2}-Bcos\left( (\omega - \omega _0)T \right) \end{aligned}$$

When $H_{DT}(z)=K_{DT}+B_{DT}\frac{z-1}{Tz}$, Condition (3-4) is equivalent to

19 $$\begin{aligned} b+B_{CT} > \frac{K_{DT}T}{2}-B_{DT}cos\left( (\omega - \omega _0)T \right) \end{aligned}$$

The frequency $\omega - \omega _0$ is arbitrary, so $cos\left( (\omega - \omega _0)T \right) \in (-1,1)$, and the most unfavourable situation of Inequality (18) occurs when $cos\left( (\omega - \omega _0)T \right) =-1$:

20 $$\begin{aligned} b+B_{CT} > \frac{K_{DT}T}{2}+B_{DT} \end{aligned}$$

In other words, if the system parameters meet the conditions of Inequalities (19) and (20),$G_1(z)$ belongs to a strictly positive real matrix, and the haptic virtual surgery simulation system is stable. Therefore, for a haptic virtual tele-operation simulation system based on the hybrid method of FPAA analogue and digital control, the complete stability condition is as follows:

21 $$\begin{aligned} b+B_{CT} > \frac{K_{DT}T}{2}+B_{DT} \end{aligned}$$

If the value of *Z*[*v*/*s*] is estimated by the backward difference method, that is, the same reasoning process is used for $G_2^{-1}(z)$ in Eq. (16), when $H_{DT}(z)=K_{DT}+B_{DT}(z-1/Tz)$ , the complete stability condition can be obtained as follows:

22 $$\begin{aligned} b + {B_{CT}} + {B_{DT}}{{ + }}\frac{{{K_{DT}}T}}{2} > 0 \end{aligned}$$

If the Tustin difference method is used to estimate the value of *Z*[*v*/*s*] , i.e., $G_3^{-1}(z)$ undergoes the same reasoning process, when $H_{DT}(z)=K_{DT}+B_{DT}(z-1/Tz)$ , the complete stability condition is as follows:

23 $$\begin{aligned} b+B_{CT}+B_{DT} > 0 \end{aligned}$$

Equations (21)–(23) indicate that the most unfavourable and conservative stability situation occurs when the forward difference method is used. Therefore, Inequality (21) is the final complete stability condition when the terminal is passive and the communication network has no delay. Therefore, only the results from the forward difference estimation method are listed hereinafter.

#### System stability inequality in the passive, delayed scenario

The stability condition obtained in “System stability inequality in the passive, undelayed scenario” is based on the premise that the operator and object are both passive and the communication network has no delay. In this section, the stability condition inequality is derived for the same haptic virtual surgery simulation system but with a delay.

Here, the system stability condition varies as the virtual environment model changes. Only the results from the forward difference estimation method are described here (as shown in the last section, forward difference estimation could obtain the limit of the stability condition). The expression from *f* to *v* to *z* domain is as follows:

24 $$\begin{aligned} \begin{array}{l} {F_h}\left( z \right) = \left( {b{{ + }}{B_{CT}}} \right) {V_h}\left( z \right) + {z^{ - n}}\left( {K + \frac{{B\left( {z - 1} \right) }}{{Tz}}} \right) \frac{T}{{z - 1}}{V_h}\left( z \right) = G_{}^{ - 1}\left( z \right) {V_h}\left( z \right) \end{array} \end{aligned}$$

Here, ${G^{ - 1}}\left( z \right) = \left( {b{{ + }}{B_{CT}}} \right) + {z^{ - n}}\left( {K + \frac{{B\left( {z - 1} \right) }}{{Tz}}} \right) \frac{T}{{z - 1}}$ . The strict positive realness of G(z) is equivalent to the strict passivity of $G^ {-1}(z)$ ; hence, the strict positive realness of $G^ {-1}(z)$ is essential^37^. Therefore, similar to 2.2.1, to ensure system stability, it is necessary to verify the positive realness of $G^ {-1}(z)$ according to Definition Appendix A-3. Condition 1 requires that all poles of $G^ {-1}(z)$ are on or within the unit circle of *z* . Equation (23) indicates that $G^ {-1}(z)$ has two poles, one at zero and the other at $z=1$ , thus satisfying Condition 1. According to Condition 3, the residue matrix of the pole corresponding to $z=1$ must have positive definiteness to ensure the strict positive realness of $G^ {-1}(z)$ . The residue obtained from (24) is as follows:

25 $$\begin{aligned} R_{01}=K_{DT}\cdot {T} \end{aligned}$$

Because $K_{m\_DT},K_{s\_DT},T > 0$, the result of (25) is a positive real number. Now, Condition 2 in Definition Appendix A-3 remains to be verified that requires ${G^{{{ - }}1}}({e^{j\left( {\omega - {\omega _0}} \right) T}}) + {G^{{{ - }}T}}({e^{ - j\left( {\omega - {\omega _0}} \right) T}})$ to be a positive real number.

If the value of $\mathrm{Z}\left[ {\frac{V}{s}} \right]$ is estimated by the forward difference method, i.e., $z = \cos (\left( {\omega - {\omega _0}} \right) T) + j\sin (\left( {\omega - {\omega _0}} \right) T)$ is substituted in Equation (24), Condition 2 is transformed to the sum of $G_1^{ - 1}({e^{j\left( {\omega - {\omega _0}} \right) T}}) + G_1^{ - T}({e^{ - j\left( {\omega - {\omega _0}} \right) T}})$ that is required to be a positive real number. According to Definition Appendix A-2, it is necessary to meet the following criterion:

26 $$\begin{aligned} 2b + 2{B_{CT}} - {K_{DT}}T + 2{B_{DT}}\cos (\left( {\omega - {\omega _0}} \right) T) - KTS > 0 \end{aligned}$$

where $S = \frac{{\sin \left( {\left( {\omega - {\omega _0}} \right) {t_d}} \right) \sin \left( {\left( {\omega - {\omega _0}} \right) T} \right) }}{{\left( {1 - \cos \left( {\left( {\omega - {\omega _0}} \right) T} \right) } \right) }}$ . When $\frac{{{t_d}}}{T} = n$ is a positive integer and ${H_{DT}}\left( z \right) = {K_{DT}} + {B_{DT}} \cdot \frac{{z - 1}}{{Tz}}$ , the worst case of Inequality (26) occurs when value is maximum. By solving $\frac{d}{{d\left( {\omega - {\omega _0}} \right) }}S = 0$ , when $\cos (\left( {\omega - {\omega _0}} \right) T) \rightarrow 1$ , the maximum value of *S* is found by detecting the sign of the second derivative of *S* . Therefore, the maximum value of *S* is as follows:

27 $$\begin{aligned} \begin{array}{l} \mathop {\lim }\limits _{\cos \left( {\left( {\omega {{ - }}{\omega _0}} \right) T} \right) \rightarrow 1} KTS= \mathop {\lim }\limits _{\cos \left( {\left( {\omega {{ - }}{\omega _0}} \right) T} \right) \rightarrow 1} KT\frac{{\sin \left( {\left( {\omega {{ - }}{\omega _0}} \right) {t_d}} \right) \sin \left( {\left( {\omega {{ - }}{\omega _0}} \right) T} \right) }}{{\left( {1 - \cos \left( {\left( {\omega {{ - }}{\omega _0}} \right) T} \right) } \right) }} = 2K{t_d} \end{array} \end{aligned}$$

So, Inequality (26) can be simplified as follows:

28 $$\begin{aligned} 2b + 2{B_{CT}} + 2{B_{DT}} - {K_{DT}}T - 2{K_{DT}}{t_d} > 0 \end{aligned}$$

In other words, if the system parameters meet the conditions of Inequalities (27) and (28), ${G_1}(z)$ is strictly positive real and the haptic virtual surgery simulation system is stable. When the terminal is passive and the delay is present in the communication network, the complete stability condition of the single-user haptic virtual surgery simulation system under hybrid control becomes as follows:

29 $$\begin{aligned} b + {B_{CT}} + {B_{DT}} > \frac{{{K_{DT}}T}}{2} + {K_{DT}}{t_d} \end{aligned}$$

#### System stability inequality in the active, undelayed scenario

The last two sections have discussed the cases of passive operators, and this section discusses active scenarios for the single-user haptic virtual surgery simulation system under hybrid control. First, the situation without system delay is considered.

In Fig. 3, for simplification, the mass *m* of the human-computer interaction device is moved to the operator impedance ${Z_h}\left( s \right)$ side. This process does not affect the transfer matrix of the overall system or the passivity of the new operator ${Z_h}\left( s \right) + ms{{ + }}\frac{{{K_{CT}}}}{s}$ . If the terminal is allowed to be active, sufficient damping needs to be moved to the operator’s side. If the actual impedance of the named operator is $- {z_a}$ , when ${z_a} > 0$ , the terminal is active. $z_a$ units of damping *b* in the human-computer interaction device (leader robot) is moved to the operator’s side, turning the new terminal to be passive. According to Inequality (20), replacing *b* with $b - {z_a}$ , for the single-user haptic virtual surgery simulation system under hybrid control that has an active terminal with no network delay, the stability condition is as follows:

30 $$\begin{aligned} b - {z_a} + {B_{CT}} > \frac{{{K_{DT}}T}}{2} + {B_{DT}} \end{aligned}$$

Let ${b_d} = b - {z_a}$ , the complete stability condition for the passive, delayed hybrid single-user haptic virtual surgery simulation system is obtained as follows:

31 $$\begin{aligned} {b_d} + {B_{CT}} > \frac{{{K_{DT}}T}}{2} + {B_{DT}} \end{aligned}$$

#### System stability inequality in the active, delayed scenario

The stability conditions derived in this section are for the single-user haptic virtual surgery simulation system under hybrid control that contains active terminals and has communication delay. First, the stability inequality in the active, undelayed scenario is derived, similar to that presented in “System stability inequality in the passive, delayed scenario”. Next, the condition of the active terminal is considered, that is, replacing *b* with $b-z_a$ in Inequalities (18) and (19):

32 $$\begin{aligned} 2b - 2{z_a} + 2{B_{CT}} - {K_{DT}}T + 2{B_{DT}}\cos (\left( {\omega - {\omega _0}} \right) T) > 0 \end{aligned}$$

Let ${b_d} = b - {z_a}$ , the complete stability condition in the active, delayed scenario becomes:

33 $$\begin{aligned} {b_d} + {B_{CT}} + {B_{DT}} > \frac{{{K_{DT}}T}}{2} + {K_{DT}}{t_d} \end{aligned}$$

### Method discussion

Refer to Appendix C ,similar methods like above have been used to analyze the complete stability conkditions for the multi-user haptic virtual surgery simulation system based on FPAA analogue/digital control under four different conditions as shown in Fig. 6.

Comparing Inequality (21) for the single-user haptic simulation system in the passive, undelayed scenario with Inequality (31) in the active, undelayed scenario, when the system has an active terminal, to ensure stability, the value ranges of control gain and sampling frequency are reduced. Although higher machine damping results in worse system performance, it can eliminate the effect of active operations. The same conclusion can be drawn in case of multiple users.

Compared with the previous stability conclusions, the stability conditions obtained here manage to slow down the compensation effect between the digital control gain and sampling frequency because of the analogue damping ${B_{CT}}$ . On one hand, compared with Ref.^32^, the operator can be active and the system is allowed to contain communication delays. On the other hand, compared with Ref.^36^, the hybrid control method expands the value range of the digital control stiffness ${K_{DT}}$ (proportional gain) through the analogue damping ${B_{CT}}$ . This allows the system to avoid high machine damping and eliminate the effect of active operations on system performance, thereby having better resistance against interferences. Therefore, from the theoretical perspective of stability, the hybrid controller is better than the single control method.

## Experiment Platform

### Construction of the haptic virtual surgery simulation platform based on FPAA analogue/digital hybrid control

To analyse the feasibility and stability of the proposed control method, it was embedded in the virtual abdominal MIS robot platform that was independently developed in our laboratory. On the simulation platform, the operator controls the tissue model in the virtual simulation environment through a human-computer interaction device and realises real-time feedback of graphics, force, and other information^38^. The proposed method was applied to the MIS simulation platform for the first time. By analysing stability and transparency, the feasibility of this control method in haptic virtual surgery simulation was demonstrated and the range of the digital control gain under stability was attained.

### Overall structure of the haptic virtual surgery simulation platform

**Figure 7:**
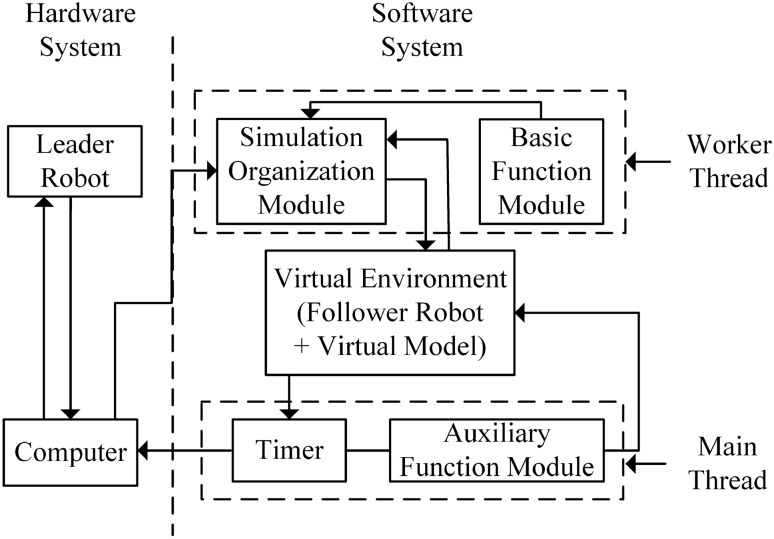
Schematic diagram of the virtual simulation platform.

The software of the virtual platform included three modules, namely, the basic function module, auxiliary function module, and operation simulation module. Two threads, the main thread and the worker thread, were present^38^. The schematic diagram of the virtual platform is shown in Fig. 7.

**Figure 8:**
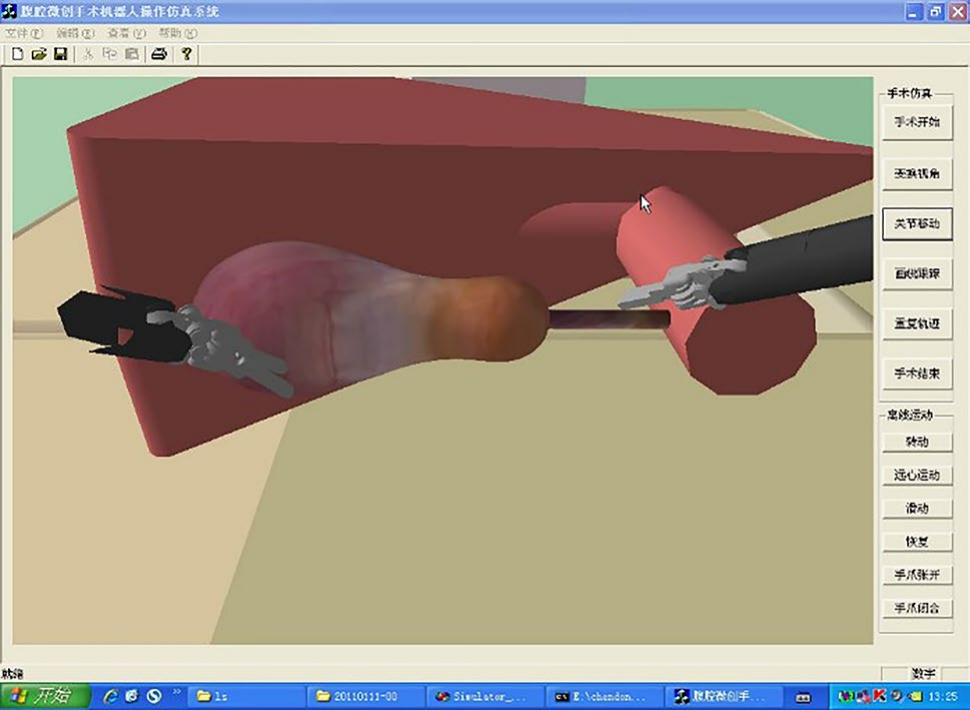
Simple graphic simulation interface.

**Figure 9:**
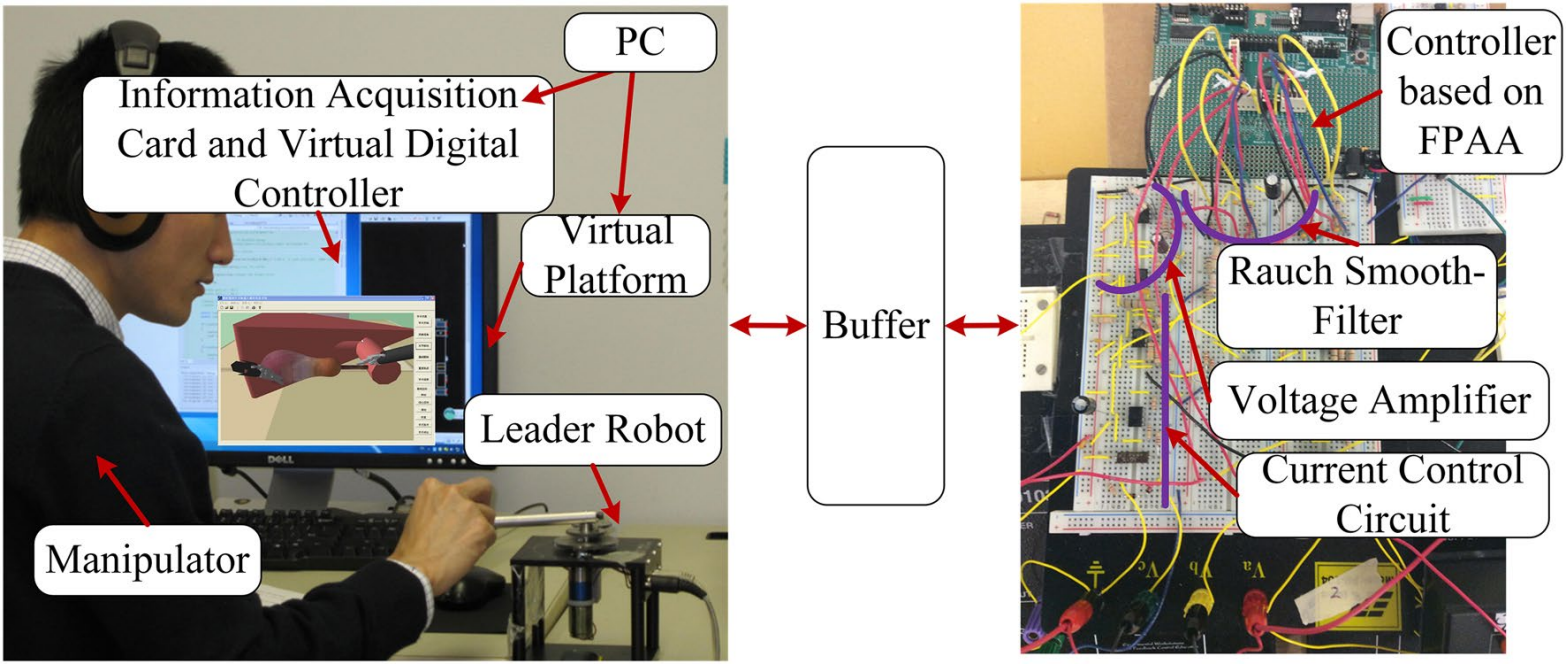
Experimental platform.

Figure 8 illustrates a simple virtual graphical simulation interface in which the leader robot is in the initial state. The experimental platform based on the virtual simulation interface is shown in Fig. 9. The computer realises the function of a virtual simulation interface and the digital controller, and performs data interaction with the human–computer interaction device (leader robot) through the data acquisition card.

When the system worked, two threads were run simultaneously. The main thread contained a common timer (SetTimer) and two multimedia timers (TimeSetEvent). The common timer was used to time the data refresh frequency, with a period of 500 ms. It is only operated when multiple virtual simulation modules interact with each other; that is, when the human–computer interaction device or the force or haptic feedback information was displayed on the interface. One of the two multimedia timers was used to time the graphics refresh frequency, with a period of 33 ms. During this time, the system only refreshed the graphics. The other multimedia timer was used to time the refresh rate of force/haptic feedback. The period was 5 ms and during this time, the system only refreshed the graphics.

All auxiliary function modules and timers were implemented in the main thread, whereas the basic function module and operation simulation module were implemented in the worker thread. Through the cooperation of the main thread and worker thread, multiple feedback refresh modules such as virtual graphics, force/touch, and simulation calculation could be realised on the virtual simulation platform. When the timer was turned on and its function was running, the corresponding modules (data refresh, force/haptic feedback, graphics refresh, etc.) in the main thread and the worker thread needed to interact with each other. To ensure data synchronisation during the interaction or data consistency, the virtual simulation platform used critical region variables for synchronisation^38^.

### Leader–follower operation control system of the haptic virtual surgery simulation platform

**Figure 10:**
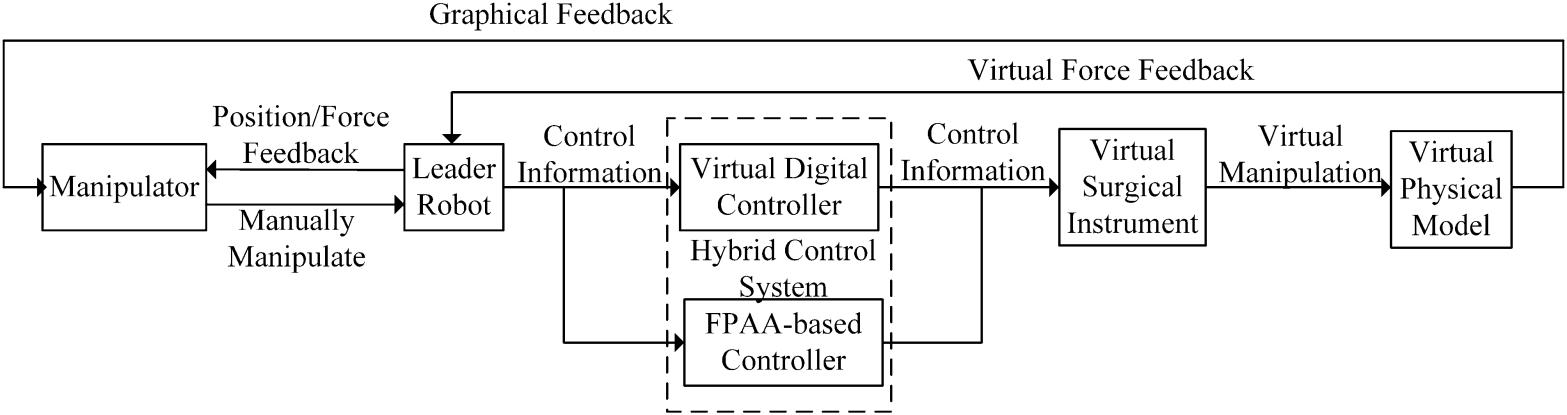
Virtual leader-follower operation system.

The virtual leader–follower operation platform independently developed by our laboratory was used, which included the operator, physical leader hand (leader robot), hybrid control system, virtual surgical instrument, and virtual physical model. The system block diagram and signal transmission are presented in Fig. 10. The FPAA-based analogue control was connected in parallel with the virtual digital control system to output the mixed control signals.

In the report by Kawai et al., to achieve more realistic force feedback, the analogue quantity was added to the leader robot arm design^39^. Here, the analogue quantity was considered as a part of the controller, which made the whole platform more flexible and allowed it to be connected with more commercial manipulators. In the subsequent simulation experiments, to simplify the experimental process, only the Servo SRV-02 fast connection module (Quanser Inc., Markham, Ontario, Canada) adopted was used as the one-dimensional rotating leader robot. The module was composed of a direct current (DC) engine, a gear, and a potentiometer. Its control current was provided by the internal current control circuit and the external displacement control circuit provided the torque command. The current control circuit was composed of only analogue components, while the external displacement control circuit was composed of the digital controller (virtual part) and the FPAA-based analogue controller. The design details of the internal current control circuit were similar to those described in Ref.^40^.

The digital signal processing in the experiment was completed by using a personal computer equipped with a 2.99 Hz dual-core Haolong processor E8400 and a 32-bit Win7 operating system. A model 826 multi-functional I/O processing card (Sensoray Co., Tigard, Oregon, USA) was used for A/D and D/A conversion. In the experiment, the displacement of the leader robot was collected by the potentiometer at the robot joint, which was then fed into the computer after A/D conversion. The force signal fed back by the virtual environment was D/A converted and the digital control signal ${F_{DT}}$ was calculated. In the meantime, the analogue controller that was parallel to the virtual environment directly received the analogue displacement information of the human-computer interaction device and calculated the analogue control signal ${F_{CT}}$ . The final ${F_{DT}} + {F_{CT}}$ was output to the leader robot. The sampling frequency during this period was the highest frequency available to the computer, namely, 1000 Hz.

**Figure 11:**
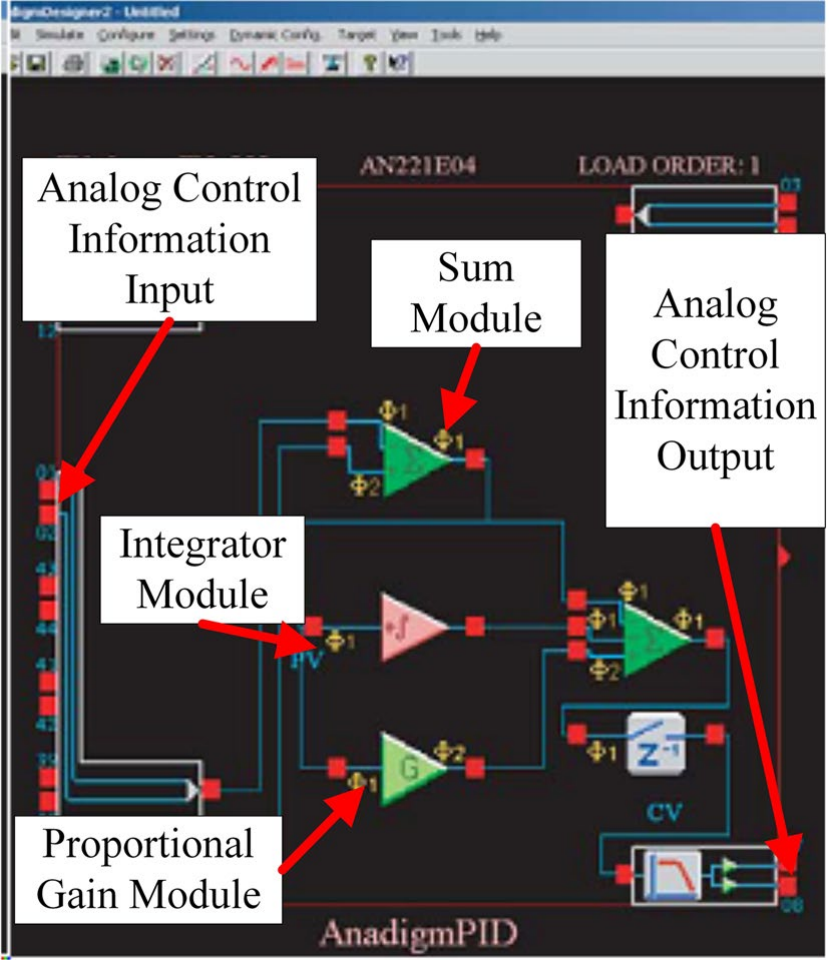
Circuit design of the PID controller.

**Figure 12:**
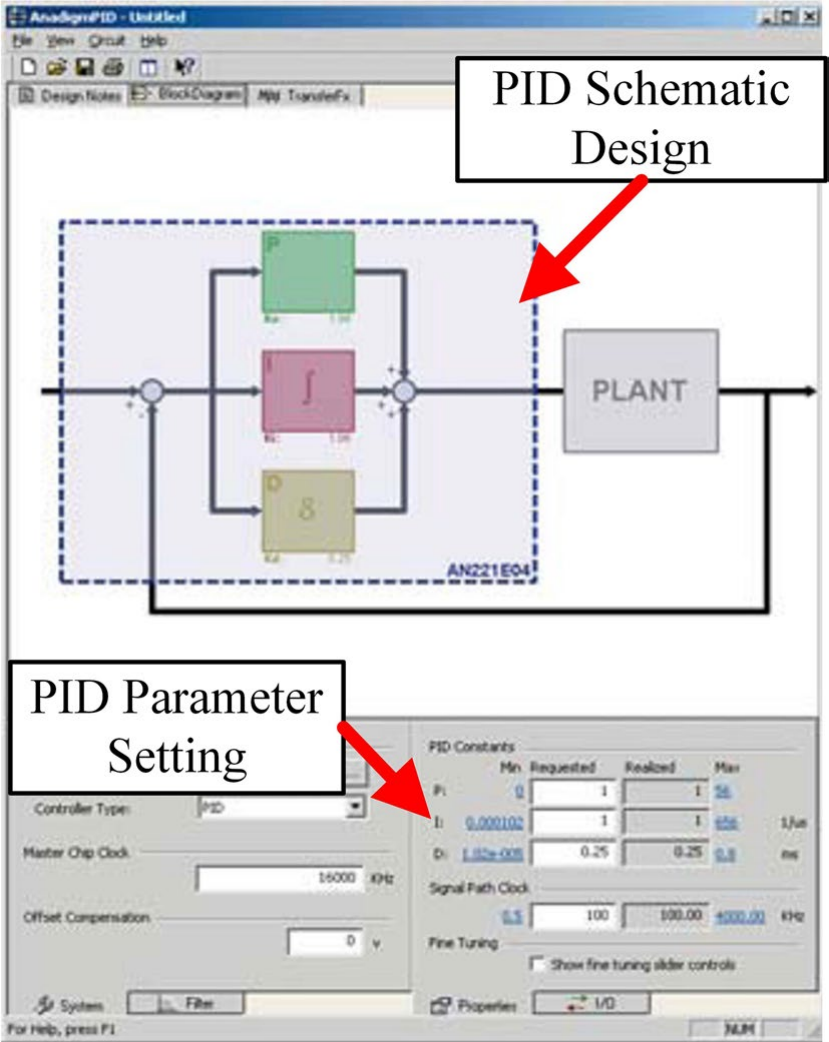
PID parameter setting interface.

Figure 11 shows the circuit diagram of the PID analogue controller. The AN221E01-series FPAA device was used and the design software was AnadigmDesigner 2.7.1. Figure 12 presents the analogue parameter setting dialog box, with the real-time error curve on the left and the PID parameter setting on the right. After determining the sampling frequency, parameter setting was completed according to the empirical trial and error method.

The input signal in Fig. 11 was the displacement information of the leader robot (i.e., the voltage read by the potentiometer of the leader robot). The analogue control information output represented the voltage output from the FPAA-based analogue PID controller after filtering, which then entered the voltage amplification module according to task needs. In Fig. 12, going through the FPAA analogue PID controller shown in Fig. 11, the transfer function of the robot controller was as follows:

34 $$\begin{aligned} {C_h} = {K_{ph}} + s \cdot {K_{dh}} + \frac{1}{s} \cdot {K_{ih}} = {K_{CT}} + s \cdot {B_{CT}} + \frac{1}{s} \cdot {I_{CT}} \end{aligned}$$

The digital controller part was located in the virtual operation simulation module. The specific model establishment and assembly process adopted the same idea and design as those in Ref.^38^.

## Results discussion

### Stability range measurement of the haptic virtual surgery simulation system under hybrid control

Through a series of experiments, the maximum stable digital control gain in the haptic virtual surgery simulation system under hybrid control was obtained when the sampling time was varied.

In the experiment, the leader robot (human–computer interaction device) was operated by the operator, and the virtual follower robot moved freely without touching any real object. The starting position of the virtual robot is also called the initial position, which might affect the results of collision detection and other behaviours. Because the stability of the passive system is independent of the initial position, initial positions with different angles were selected to eliminate their effect on the stability test.

Definition of an unstable state: In the experiment, the operator operates the leader robot and the virtual device does not contact any virtual simulation object or load. If the position of the leader robot or virtual device is not controlled or has random vibrations, the haptic virtual surgery simulation system is deemed unstable^29^. On the other hand, if the robot position is always within the boundary and exhibits no autonomous vibrations, the system is considered stable.

In the simulation control part based on FPAA, when the continuous control gain coefficient is greater than zero, the haptic virtual surgery simulation system meeting the design constraints is stable^41^. According to the selected parameters, the simulation control part based on FPAA in this paper was always in a stable state.

In the digital control part, according to the stability conditions in (20) and (32), the stability range is related to the damping of the leader-follower robot, the digital control gain, the sampling period, and the analogue damping term. In the experiment, the damping b of the robot was obtained to be $b = 0.0018\, {{\mathrm{N} \, \mathrm{s}}/ \mathrm{m}}$ by the grey rectangular system identification method^41^. Using the parameter tuning software displayed in Fig. 12, the damping (differential term) of the analogue controller could be selected as ${B_{CT}} = 0.25\,{{\mathrm{N} \, \mathrm{s}} / \mathrm{m}}$ , the proportional gain term was ${K_{CT}} = 10{{\mathrm{N} \, \mathrm{m}} / \mathrm{rad}}$ , the sampling period gradually increased from 1 ms to 10 ms, and the interval was 1 ms. The above options were ideal average parameters based on multiple experiments.

Under the selected sampling period, choose a *K* value close to the limit. If the system was still stable, maintain this *K* value and *T* value, and transform the initial position of the virtual robot. If the system was stable under all initial positions, the point *K*–*T* was located in the completely stable area of the system; otherwise, it was in an incompletely stable area. If the system at this point was completely stable, increase the value of *K* by 0.1 each time until instabilities such as vibrations occurred. Finally, the upper limit of stability for this point was obtained. It was necessary to ensure that the initial position transformation experiment was performed after each value of *K* was selected. The experimental results are shown in the form of a *K*–*T* graph, in which the maximum *K* values when the system was completely stable were marked with an asterisk at each sampling period *T* . In the same sampling period, the minimum *K* values when the system became unstable were marked with a hollow circle.

**Figure 13:**
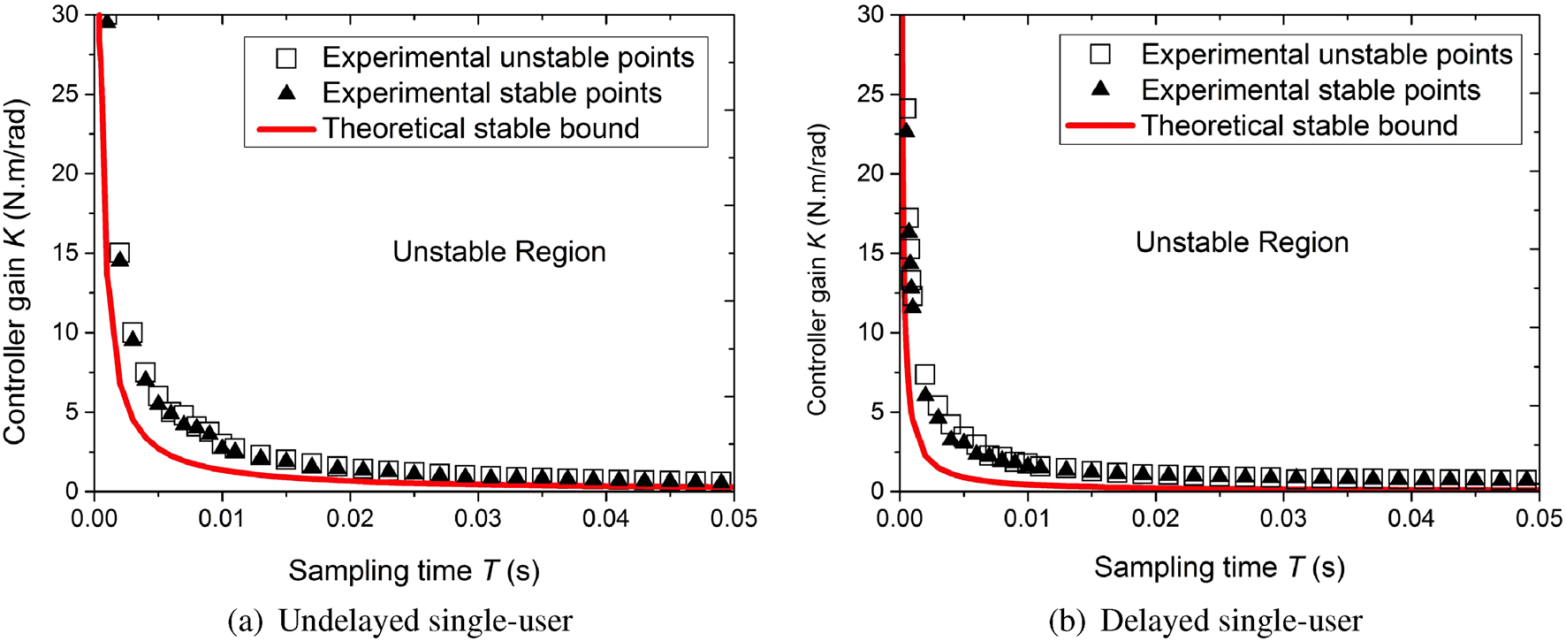
Comparison of theoretical and measured control gains in the virtual haptic surgery simulation system.

During the entire experiment, the system operated normally, and the final stability boundary is given in Fig. 13. Figure 13a corresponds to the case without delay in a passive single-user haptic virtual surgery simulation system. Figure 13b exhibits the scenario with a communication delay of ${t_d} = 5T$ . For these two cases, the stability and instability regions obtained according to (20) and (32) are distinguished by black solid lines. These results showed that for each sampling period, the theoretical value range of stability condition was more conservative than the experimental one.

### Comparative analysis of PD and PID control strategies under hybrid control

Bilateral teleoperation system is a dynamic tracking system, which requires the system output to be as close to the input as possible; that is, the amplitude and phase of the output should be as similar to the input as possible. The differential control term can improve the dynamic performance of the system and reduce the tracking error of the dynamic signal. Theoretically, the integral control term I can add a pole at the origin, which is conducive to eliminating the steady-state error. However, this may increase the phase lag and compromise system stability^41^. On the other hand, when studying the haptic virtual surgery simulation system, to indicate the existence of machine damping, the control gain is usually regarded as the sum of the damping and proportional terms^31–34^.

**Figure 14:**
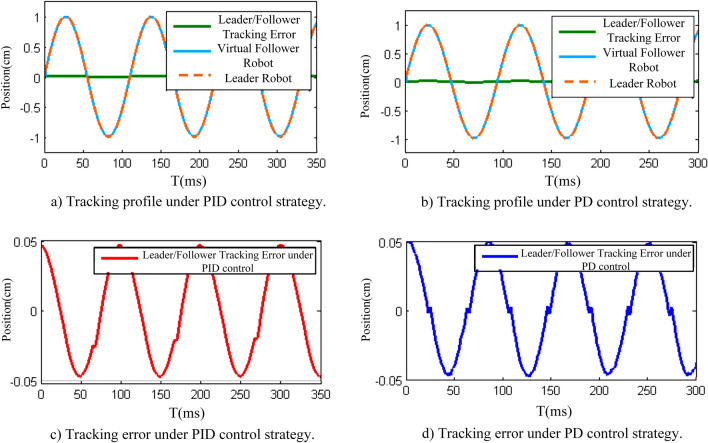
Leader–follower position tracking error profiles under different control strategies.

The posture tracking errors under PID control and PD control were compared in the free motion experiment of the haptic virtual surgery simulation system under hybrid control, and the results are presented in Fig. 14.

In Fig. 14, to eliminate the effect of human hands on the control system, the leader robot used a signal generator to input a sinusoidal signal with an amplitude of 1 and a frequency of 10 *Hz* . Here, free motion meant the moving of the leader robot by the operator when the (virtual) follower robot had no load and did not contact any operation object or environment.

In the FPAA hybrid controller, only the proportional term was used in the digital control. As can be seen from Fig. 13, in the delay-free single-user scenario, the maximum value that can be taken when the sampling period was 1 ms was $30 \, {\mathrm{N} \, \mathrm{m}}/\mathrm{rad}$ . In the FPAA control part, according to the parameter setting software displayed in Fig. 12, under the PID strategy, if the proportional gain term was selected as ${K_{CT}} = 10\,{{\mathrm{N} \, \mathrm{m}} / \mathrm{rad}}$ , the dynamic performance was the best when the integral gain term was ${I_{CT}}{{ = 0}}.29$ and the damping (differential term) was ${B_{CT}} = 0.04{{\mathrm{N} \, \mathrm{s}} / \mathrm{m}}$ . When the PD strategy was adopted, if the proportional gain term for the analogue controller was ${K_{CT}} = 10{{\mathrm{N} \, \mathrm{m}} / \mathrm{rad}}$ , the dynamic performance was optimal when the damping (differential term) was ${B_{CT}} = 0.025{{\mathrm{N} \, \mathrm{s}} / \mathrm{m}}$ .

The results demonstrated that the system poses tracking error values (Fig. 14c,d) under the two control strategies were within $\pm 0.05cm$ . On the other hand, to realise bidirectional PID control, two FPAA chips were needed to complete the circuit design, while the realisation of bidirectional PD control only needed one chip^40^. Considering the system performance and economic factors, PD control was used in the displacement control circuit in the experiment.

### Posture tracking evaluation experiment of the haptic virtual surgery simulation system under hybrid control in free motion

The ‘free motion’ in this paper means that when the slave robot has no load and does not contact any operation object/environment, the operator moves the leader robot. The system transparency was investigated by analysing the error between the virtual displacement and the displacement information of the human–computer interaction device. According to the definition of transparency, the smaller the pose tracking error, the better the transparency.

**Figure 15:**
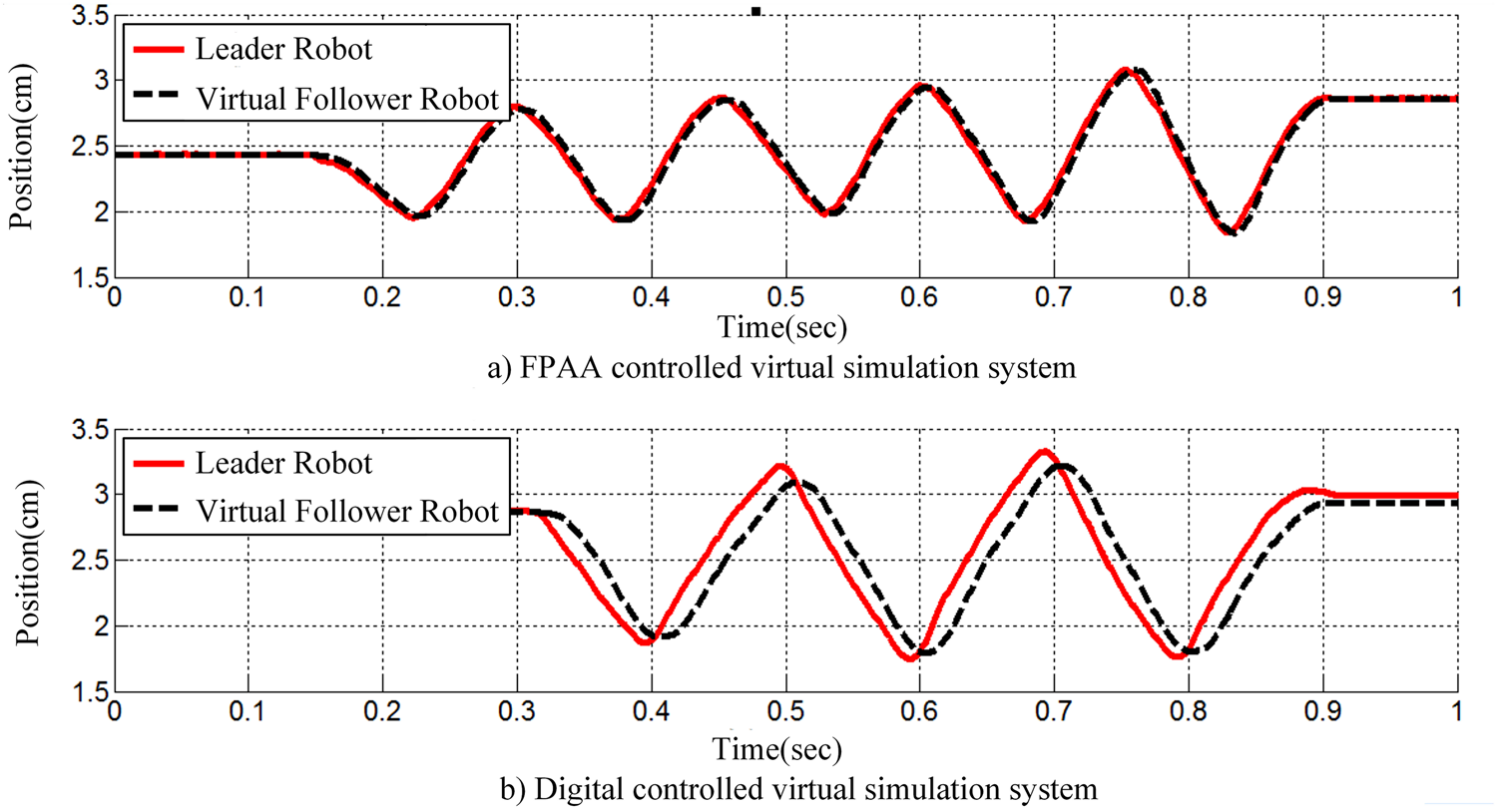
Leader–follower position tracking profiles under free motion for systems under digital and hybrid control.

The posture tracking performance of the haptic virtual surgery simulation system under hybrid control and the system using digital control alone were compared in a free motion experiment, and the results are provided in Fig. 15. According to the conclusion in Fig. 13, in the process of pose tracking, the maximum digital control gain was 30 ${{\mathrm{N} \, \mathrm{m}} / \mathrm{rad}}$ , the damping of the leader robot’s analogue controller was 0.025 ${{\mathrm{N} \, \mathrm{m}} / \mathrm{rad}}$ , and the proportional gain was 10 ${{\mathrm{N} \, \mathrm{m}} / \mathrm{rad}}$ .

In Fig. 15, the Euclidean norm of the pose tracking error under digital control was 0.042 cm, and the pose tracking error of the system under hybrid control was 0.225 cm. As can be seen from Fig. 15, with either control method, the haptic virtual surgery simulation system could reduce the pose tracking error between the leader and slave robots when increasing the control gain. However, when the gain was at its maximum, the pose difference of the system under digital control was considerably greater than that under hybrid control. This exhibited the superiority of the haptic virtual surgery simulation system under hybrid control in terms of transparency.

### Transparency evaluation experiment of force feedback in the haptic virtual surgery simulation system under hybrid control

As shown in Fig. 3, the system only had position sensors, so the force feedback was perceived by the operator according to the displacement information of the human–computer interaction device. Therefore, the transparency of force feedback could be analysed according to the displacement difference. The smaller the displacement difference, the better the transparency of force feedback^42^.

#### Design of the force feedback transparency evaluation experiment of the system under hybrid control

The effects of force feedback were analysed by identifying virtual tissues with different hardness. These object recognition experiments have many applications, such as palpation of local cancer tissues in MIS. To allow the operator to complete object hardness identification in the virtual environment, the impedance felt by the operator should be as close to the stiffness set by the virtual object as possible. Therefore, if one or two virtual objects are set with high hardness, the haptic virtual surgery simulation control system needs to provide a large impedance, which corresponds to the gain of the controller. In the experiment, the control gain of the leader robot (human–computer interaction device) needs to be sufficiently high to provide a high impedance value.

Subjects: Five participants (three men and two women).

Objective: Determine according to the virtual force feedback whether the hardness of the two virtual tissues were the same and compare their hardness grades.

Procedure: The participant first operated the human-computer interaction device to touch the virtual gallbladder A, and then touched another gallbladder tissue with the same/different hardness (hardness *A* or *B* , $A < B$ ). The participant should determine whether the tissue at the second touch was harder, softer, or had the same hardness compared to the first one. Each participant conducted a total of 18 randomly ordered experiments, with a slight interval between two successive experiments. Before the formal test, each participant could practice two to three times to adapt to the tele-operation system and understand the experiment’s objective. Each time, the participant had 30 seconds to complete the task and make judgments. The simulation experiment platform is shown in Figs. 8 and 9.

The initial gallbladder tissue parameters were consistent with those in Ref.^38^, with an initial pressure of 2.94 kPa . When the hardness was *A* , the gallbladder area pressure was 4 kPa ($4{\mathrm{kN}} / {\mathrm{m}}^2$). When the hardness was *B* , the surface pressure was 5 kPa ($5{\mathrm{kN} / {\mathrm{m}{^2}}}$) . At the contact point between the virtual device and the tissue, the *x*-axis elastic coefficient was 200 N / m , the original length of the spring was 2 mm , the damping coefficient was $200 \mathrm{N / s}$ . For the *y* -axis, the elastic coefficient was 180 N / m , the original spring length was 2 mm , the damping coefficient was 180 N /s . As for the *x* -axis, the elastic coefficient was 10*N*/*m* , the original spring length was 2*mm* , and the damping coefficient was 10 N / s . The contact area was a circular gallbladder tissue surface with a diameter of 10 mm .

The special points here were as follows. (1) The slave robot was completely realised in the virtual environment, and the leader robot was a human-computer interaction device. The parameter settings of the virtual slave robot were consistent with those of the human–computer interaction device (Servo SRV-02 quick connection module, Ouanser Inc., Markham, Ontario, Canada). (2) The identified object was the gallbladder model simulated in Fig. 8, and two different hardnesses ( *A* or *B* , $A < B$ ) were set. (3) Two different controllers (hybrid or digital) and three test scenarios (AA, BB, or AB/BA) were considered in the experiment.

#### Transparency experiment result and discussion

**Figure 16:**
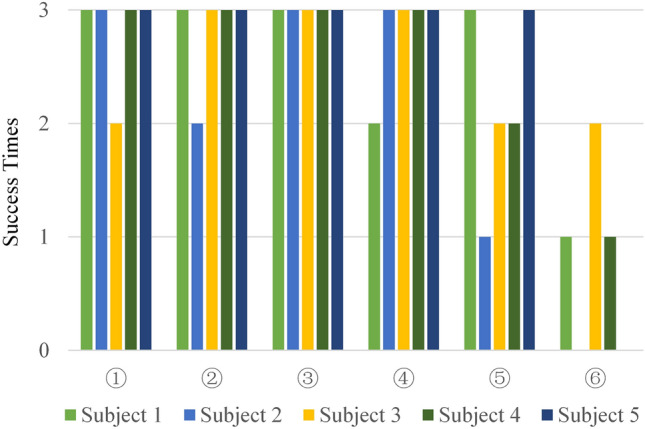
Task success results under the two control conditions.

Figure 16 presents the success rates in the 18 tests for each of the five operators in the form of a chart. In Fig. 16, orange represents Participant 1, light blue Participant 2, purple Participant 3, brown Participant 4, and dark blue Participant 2. References ①,③,⑤ correspond to the hybrid control results whereas②,④,⑥ correspond to the cases with a digital controller. In cases①,②, the operator touched soft test objects (AA) twice. In cases③,④, the operator touched hard test objects (BB) twice. In cases⑤,⑥, the operator touched two test objects with different hardness (AB/BA) in random order. Each subject tested three times for each control case. As shown in Fig. 16, when the hybrid method was used, the task success times were higher. In other words, the impedance intensity perceived by the participants was greater and the system exhibited better transparency.

The statistical significance of the results under the two controllers was studied by the unilateral T-test^43^, and the results are presented in Table 1. The T-test p-value between①,② was equal to the statistical threshold (0.05), indicating that when soft gallbladder tissues (AA) were touched twice, no significant numerical difference existed in the results using the two different controllers. In other words, both controllers could successfully assist participants to achieve a high success rate. The p-value between③,④ was 0.18695, indicating a significant difference in the experimental results of the BB scenario. The p-value of ⑤,⑥ was $p = 0.01722$ , which indicated a significant difference in the statistical results between the two groups. In other words, when distinguishing two test objects with different hardness (AB/BA), the experimental results using the hybrid controller were better.

According to the experimental results, the two controllers could well identify the soft gallbladder tissues with a hardness of A. However, when identifying the hard tissues B, the simulation results using the hybrid controller were considerably better than those using a digital controller alone. The experimental results demonstrated that the virtual simulation system under hybrid control was better than that under digital control in transmitting task-related information (e.g., transmission impedance).

**Table 1:** Right-tailed T-test results under different control conditions.

Scenario	Condition					
	①	②	③	④	⑤	⑥
	Using a hybrid controller to touch AA tissues	Using a digital controller to touch AA tissues	Using a hybrid controller to touch BB tissues	Using a digital controller to touch BB tissues	Using a hybrid controller to touch AB/BA tissues	Using a digital controller to touch AB/BA tissues
①	–	–	–	–	–	–
②	0.05	–	–	–	–	–
③	0.18695	0.18695	–	–	–	–
④	0.68695	0.50000	0.18695	–	–	–
⑤	0.04484	0.01722	0.01670	0.07067	–	–
⑥	0.03473	0.00162	0.00211	0.00544	0.01722	–

## Conclusion

Using FPAA-based analogue control to reduce the effect of controller discretisation on the transparency of the virtual bilateral system, this paper realised the hybrid control method in the haptic virtual surgery simulation system, deduced its stability conditions, and expanded the impedance range under stability. The stability conditions considered four cases: passive and undelayed, passive and delayed, active and undelayed, and active and delayed. The single-user conclusions were also extended to the cases of multiple users. Through the simulation platform independently developed in our laboratory, the value range of the proportional gain under stable conditions was determined. The transparency evaluation experiment demonstrated that the haptic virtual surgery simulation system under hybrid control had better force feedback performance in hard object detection.

Considering the results and experimental platform used in this study, the following work needs to be performed in the future. (1) This paper adopted the position difference-based process environment block (PEB) tele-operation structure that had optimal stability. Further, the performance of a four-channel tele-operation system can be investigated to complete the stability conditions of the hybrid control method. (2) Another focus can be on FPAA-based programming to realise the functions such as dynamic continuous damping and proportional gain transformation.

## Supplementary Information

Supplementary Information.
