# Supplementary material for: Haptic virtual surgery simulation system under field programmable analogue array-based hybrid control
Source: Sci Rep. 2022 Jul 20;12:12371. doi: 10.1038/s41598-022-16655-9 (PMC9300654; doi:10.1038/s41598-022-16655-9)
Supplement: Supplementary file 1 — Supplementary Information. [file 41598_2022_16655_MOESM1_ESM.pdf]

Appendices

A. Fundamentals of Mathematics

This appendix provides the mathematical theoretical basis used for stability analysis in the present study.

Definition Appendix A-1: If A is a Hermitian symmetric matrix, then this matrix is a semi-positive definite matrix (positive definite matrix) when all its principal minors are non-negative (positive matrices).

Definition Appendix A-2[1]: If $u^T y \geq 0$, then $y = h(t,u)$ is a passive system. Otherwise, the system is active.

Definition Appendix A-3[1]: A $m \times m$ regular rational function matrix $G(z)$ is a positive real matrix when the following conditions are met:

(1) All the poles of $G(z)$ are inside or on the unit circle;

(2) For any real number ω , $e^{j\omega T}$ is not a pole of $G(z)$, and the corresponding matrix $G\left(e^{j\omega T}\right) + G^T\left(e^{-j\omega T}\right)$ is a semi-positive definite matrix;

(3) The poles of $G(z)$ on $|z| = 1$ are simple poles, and the corresponding residue matrices are semi-positive definite matrices.
The transfer function $G(z)$ is a positive real number if $\omega_0 > 0$, which makes $G(e^{j(\omega-\omega_0)T})$ a positive real number.

Theorem Appendix A-1[1]: Minimum implementation problems for linear time invariant systems (LTI) can be described as follows:

$$x(i+1) = Ax(i) + Bu(i) \tag{AppendixA-1}$$

$$y(i) = Cx(i) + Du(i) \tag{AppendixA-2}$$

Here, when $G(z)$ is a positive real matrix (strictly positive real matrix), the $G(z) = C(zI-A)^{-1}B + D$ is passive (strictly passive).
Theorem Appendix A-2[2]: In the discrete multivariable control system as shown in Definition Appendix A-1, wherein the forward channel is a linear time-invariant (LTI) system, with a nonlinear feedback channel and a transfer function $\varphi = \varphi(y)$, the system can be expressed as follows:

$$x(i+1) = Ax(i) - B\varphi(y) \tag{AppendixA-3}$$

$$y(i) = Cx(i), \quad y \in R^m \tag{AppendixA-4}$$

$$\varphi(y) = [\varphi_1(y_1),\ \varphi_2(y_2),\ \ldots,\ \varphi_m(y_m)]^T \tag{AppendixA-5}$$

For any passive function $\varphi(y)$, the system (Appendix A–3) to (Appendix A–5) is stable when $C(zI-A)^{-1}B$ is a positive real matrix (according to Theorem Appendix A-1, the function is strictly passive, i.e. $G(z)$ is a positive real matrix).

B. Difference estimation methods

This study used the following three difference estimation methods, which have been used in various bilateral control studies:

Forward difference estimation method:

$$x_h(kT+K) = x_h(kT) + T\overset{\bullet}{x}_h(kT) \xrightarrow{Z} zX_h(z) = X_h(z) + TV_h(z) \Rightarrow X_h(z) = \frac{T}{z-1}V_h(z) \tag{AppendixB-1}$$

Backward difference estimation method:

$$x_h(kT) = x_h(kT-T) + T\overset{\bullet}{x}_h(kT) \xrightarrow{Z} X_h(z) = z^{-1}X_h(z) + TV_h(z) \Rightarrow X_h(z) = \frac{Tz}{z-1}V_h(z) \tag{AppendixB-2}$$

Tustin deformation method:

$$\begin{aligned} x_h(kT+T) &= x_h(kT) + T\overset{\bullet}{x}_h(kT) + \left(\overset{\bullet}{x}_h(kT+T) - \overset{\bullet}{x}_h(kT)\right)\frac{T}{2} \xrightarrow{Z} zX_h(z) = X_h(z) + TV_h(z) + (zV_h(z) - V_h(z))\frac{T}{2} \\ &\Rightarrow X_h(z) = \frac{T}{2}\frac{z+1}{z-1}V_h(z) \end{aligned} \tag{AppendixB-3}$$

C.Stability analysis of the Multi-user haptic virtual surgery simulation system

This appendix presents the complete stability conditions for the multi-user haptic virtual surgery simulation system based on FPAA analogue/digital control under four different conditions as shown in Section 2.1.

C-1 System stability inequality in the passive, undelayed scenario

When the operators are passive, according to the multivariate discrete circle criterion, i.e., Theorem Appendix A-2, to ensure system stability, $G(z)$ needs to be a positive real matrix. Similar to the analysis for a single-user system, the passivity of $G(z)$ is equivalent to the passivity of $G^{-1}(z)$, and the positive realness of $G^{-1}(z)$ ensures that $G(z)$ is also positive real. $G(z)$ is a $m \times m$ matrix, so the case of $m = 2$ can be considered first.

$$G^{-1}(z) = \begin{bmatrix} \begin{bmatrix} (b_1 + B_{1CT}) \\ + \frac{(K_{01DT} + K_{12DT})T}{z-1} \\ + \frac{(B_{01DT} + B_{12DT})}{z} \end{bmatrix} & -\left(\frac{K_{12DT}T}{z-1} + \frac{B_{12DT}}{z} \right) \\ -\left(\frac{K_{12DT}T}{z-1} + \frac{B_{12DT}}{z} \right) & \begin{bmatrix} (b_2 + B_{2CT}) \\ + \frac{(K_{02DT} + K_{12DT})T}{z-1} \\ + \frac{(B_{02DT} + B_{12DT})}{z} \end{bmatrix} \end{bmatrix} \tag{AppendixC-1}$$

To ensure system stability, verifying the positive realness of $G^{-1}(z)$ according to Definition Appendix A-3 is necessary. Condition 1 requires that all poles of $G^{-1}(z)$ must be on or within the unit circle. According to Equation (Appendix C-1), $G^{-1}(z)$ has two poles, one at zero and the other on $z = 1$, thus satisfying Condition 1. According to Condition 3, the residue matrix of the pole corresponding to $z = 1$ must have positive definiteness to ensure the strict positive realness of $G^{-1}(z)$. The residue matrix obtained from (Appendix C-1) is:

$$R_0 = \begin{bmatrix} (K_{01DT} + K_{12DT}) \cdot T & -K_{12DT} \cdot T \\ -K_{12DT} \cdot T & -(K_{02DT} + K_{12DT}) \cdot T \end{bmatrix} \tag{AppendixC-2}$$

Because $K_{01DT}, K_{02DT}, K_{12DT}, T > 0$ and $\det(R_0) > 0$, as can be known from Definition Appendix A-1, Matrix (Appendix C-2) is a positive definite matrix. The remaining Condition 2 demands that $G^{-1}(e^{j(\omega-\omega_0)T}) + G^{-T}(e^{-j(\omega-\omega_0)T})$ is a positive definite matrix.

If the value of $\mathrm{Z}\left[\frac{V}{s}\right]$ is estimated by the forward difference method, i.e., $z = \cos((\omega-\omega_0)T) + j\sin((\omega-\omega_0)T)$ is substituted in Equation (Appendix C-1), Condition 2 is transformed such that the sum of $G^{-1}(e^{j(\omega-\omega_0)T}) + G^{-T}(e^{-j(\omega-\omega_0)T})$ is required to be a positive definite matrix. According to Definition Appendix A-2, it is required to meet:

$$2(b_1 + B_{1CT}) - (K_{01DT} + K_{12DT})T + 2(B_{01DT} + B_{12DT})\cos((\omega-\omega_0)T) > 0 \tag{AppendixC-3}$$

$$\begin{aligned} &\det\left(G^{-1}(e^{j(\omega-\omega_0)T}) + G^{-T}(e^{-j(\omega-\omega_0)T})\right) \\ &= (2b_1 + 2B_{1CT} - K_{01DT}T + 2B_{01DT}\cos((\omega-\omega_0)T)) \cdot (2b_2 + 2B_{2CT} - K_{02DT}T + 2B_{02DT}\cos((\omega-\omega_0)T)) \\ &\quad - \begin{bmatrix} (K_{01DT} + K_{02DT})T - (2b_1 + 2B_{1CT} + 2b_2 + 2B_{2CT}) \\ -2(B_{01DT} + B_{02DT})\cos((\omega-\omega_0)T) \end{bmatrix} \cdot (2B_{12DT}\cos((\omega-\omega_0)T) - K_{12DT}T) > 0 \end{aligned} \tag{AppendixC-4}$$

When $C_{iDT}(z) = K_{DT} + B_{DT} \cdot \frac{z-1}{Tz}, \quad i = 1, \cdots, m$, because the frequency $\omega - \omega_0$ is arbitrary, $\cos((\omega-\omega_0)T) \in (-1, 1)$. So, when $\cos((\omega-\omega_0)T) = -1$, the most unfavourable situation of Inequalities (Appendix C-3) and (Appendix C-4) occurs:

$$\left(b + B_{CT} - \frac{K_0T}{2} - B_{0DT}\right)^2 > \left(b + B_{CT} - \frac{K_0T}{2} - B_{0DT}\right)(K_{12DT}T + B_{12DT}) \tag{AppendixC-5}$$

Here, $b = \min(b_1, b_2)$, $B_{CT} = \min(B_{1CT}, B_{2CT})$, $B_{0DT} = \min(B_{01DT}, B_{02DT})$, and $K_0 = \min(K_{01DT}, K_{02DT})$.

By simplifying (Appendix C-5), the stability condition of the bilateral haptic virtual surgery simulation system can be obtained as follows:

$$b + B_{CT} > \frac{K_{0DT}T}{2} + K_{12DT}T + B_{0DT} + 2B_{12DT} \tag{AppendixC-6}$$

Similarly, when $m=3$, $G^{-1}(z)$ becomes:

$$G^{-1}(z)=\begin{bmatrix} \begin{bmatrix} (b_1+B_{1CT}) \\ +\frac{K_{1DT}T}{z-1}+\frac{B_{1DT}}{z} \end{bmatrix} & -\left(\frac{K_{12DT}T}{z-1}+\frac{B_{12DT}}{z}\right) & -\left(\frac{K_{13DT}T}{z-1}+\frac{B_{13DT}}{z}\right) \\ -\left(\frac{K_{12DT}T}{z-1}+\frac{B_{12DT}}{z}\right) & \begin{bmatrix} (b_2+B_{2CT}) \\ +\frac{K_{2DT}T}{z-1}+\frac{B_{2DT}}{z} \end{bmatrix} & -\left(\frac{K_{23DT}T}{z-1}+\frac{B_{23DT}}{z}\right) \\ -\left(\frac{K_{13DT}T}{z-1}+\frac{B_{13DT}}{z}\right) & -\left(\frac{K_{23DT}T}{z-1}+\frac{B_{23DT}}{z}\right) & \begin{bmatrix} (b_3+B_{3CT}) \\ +\frac{K_{3DT}T}{z-1}+\frac{B_{3DT}}{z} \end{bmatrix} \end{bmatrix} \quad \text{(AppendixC-7)}$$

Here, $K_{1DT}=K_{01DT}+K_{12DT}+K_{13DT}$, $K_{2DT}=K_{02DT}+K_{12DT}+K_{23DT}$, $K_3=K_{03DT}+K_{13DT}+K_{23DT}$, $B_{1DT}=B_{01DT}+B_{12DT}+B_{13DT}$, $B_{2DT}=B_{02DT}+B_{12DT}+B_{23DT}$, $B_{3DT}=B_{03DT}+B_{13DT}+B_{23DT}$. As explained above, $G^{-1}(z)$ needs to be a positive real matrix. According to Equation (Appendix C-7), $G^{-1}(z)$ has two poles, one at zero and the other on $z=1$, which meets Condition 1 of Definition Appendix A-3. According to Condition 3, the residue matrix of the pole corresponding to $z=1$ must have positive definiteness to ensure the strict positive realness of $G^{-1}(z)$. The residue matrix obtained from (Appendix C-7) is as follows:

$$R_0=\begin{bmatrix} \left[\begin{pmatrix} K_{01DT} \\ +K_{12DT} \\ +K_{13DT} \end{pmatrix}\cdot T\right] & -K_{12DT}\cdot T & -K_{13DT} \\ -K_{12DT} & \left[\begin{pmatrix} K_{02DT} \\ +K_{12DT} \\ +K_{23DT} \end{pmatrix}\cdot T\right] & -K_{23DT} \\ -K_{13DT} & -K_{23DT} & \left[\begin{pmatrix} K_{03DT} \\ +K_{13DT} \\ +K_{23DT} \end{pmatrix}\cdot T\right] \end{bmatrix} \quad \text{(AppendixC-8)}$$

where $K_{ijDT}, T>0, i=0,\cdots,2, j=1,\cdots,3$, and $\det(R_0)>0$.

Therefore, Matrix (Appendix C-8) is a positive definite matrix that satisfies Condition 3. Condition 2 also needs to be met; that is, $G^{-1}(e^{j(\omega-\omega_0)T})+G^{-T}(e^{-j(\omega-\omega_0)T})$ is required to be a positive definite matrix.

By substituting $z=\cos((\omega-\omega_0)T)+j\sin((\omega-\omega_0)T)$ into Equation (Appendix C-7), Condition 2 of Definition Appendix A-3 is transformed such that the sum of $G_1^{-1}(e^{j(\omega-\omega_0)T})+G_1^{-T}(e^{-j(\omega-\omega_0)T})$ should be a positive definite matrix. According to Definition Appendix A-2, the following condition is required to be met:

$$b_1+B_{1CT}>\frac{(K_{01DT}+K_{12DT}+K_{13DT})T}{2}+(B_{01DT}+B_{12DT}+B_{13DT})\cos((\omega-\omega_0)T) \quad \text{(AppendixC-9)}$$

$$\begin{aligned} &\det\left(G^{-1}(e^{j(\omega-\omega_0)T})+G^{-T}(e^{-j(\omega-\omega_0)T})\right) \\ &=(2b_1+2B_{1CT}-(K_{01DT}+K_{12DT}+K_{13DT})T+2(B_{01DT}+B_{12DT}+B_{13DT})\cos((\omega-\omega_0)T)) \\ &\quad\cdot(2b_2+2B_{2CT}-(K_{02DT}+K_{12DT}+K_{23DT})T \\ &\quad+2(B_{02DT}+B_{12DT}+B_{23DT})\cos((\omega-\omega_0)T))\cdot(2b_3+2B_{3CT}-(K_{03DT}+K_{13DT}+K_{23DT})T \\ &\quad+2(B_{03DT}+B_{13DT}+B_{23DT})\cos((\omega-\omega_0)T))-2(K_{12DT}T+2B_{12DT})(K_{13DT}T+2B_{13DT})(K_{23DT}T+2B_{23DT}) \\ &\quad-\begin{bmatrix} 2b_1+2B_{1CT}-(K_{01DT}+K_{12DT}+K_{13DT})T \\ +2(B_{01DT}+B_{12DT}+B_{13DT})\cos((\omega-\omega_0)T) \end{bmatrix}\cdot(K_{23DT}T+2B_{23DT})^2 \\ &\quad-\begin{bmatrix} 2b_2+2B_{2CT}-(K_{02DT}+K_{12DT}+K_{23DT})T \\ +2(B_{02DT}+B_{12DT}+B_{23DT})\cos((\omega-\omega_0)T) \end{bmatrix}\cdot(K_{13DT}T+2B_{13DT})^2 \\ &\quad-\begin{bmatrix} 2b_3+2B_{3CT}-(K_{03DT}+K_{13DT}+K_{23DT})T \\ +2(B_{03DT}+B_{13DT}+B_{23DT})\cos((\omega-\omega_0)T) \end{bmatrix}\cdot(K_{12DT}T+2B_{12DT})^2>0 \end{aligned}$$

(AppendixC-10)

When $C_{iDT}(z)=K_{DT}+B_{DT}\cdot\frac{z-1}{Tz},\quad i=1,\cdots,m$, because the frequency $\omega-\omega_0$ is arbitrary, $\cos((\omega-\omega_0)T)\in(-1,1)$. Therefore, the most unfavourable situation of Inequalities (Appendix C-9) and (Appendix C-10) occurs when $\cos((\omega-\omega_0)T)=-1$:

$$(2b+2B_{CT}-K_{0DT}T-2B_{0DT}-4B)^3-2(K_{DT}T+2B_{DT})^3>3(K_{DT}T+2B)^2(2b-K_{0DT}T-2B_{0DT}-4B_{DT})$$

(AppendixC-11)

Here, $K_{0DT}=\min(K_{01DT},K_{02DT},K_{03DT})$, $K_{DT}=\min(K_{12DT},K_{13DT},K_{23DT})$, $B_{0DT}=\min(B_{01DT},B_{02DT},B_{03DT})$, $B_{DT}=\min(B_{12DT},B_{13DT},B_{23DT})$, $b+B_{CT}=\min(b_1{+}B_{1CT},b_2{+}B_{2CT},b_3{+}B_{3CT})$. By simplifying (Appendix C-5), the stability condition of the three-user haptic virtual surgery simulation system can be obtained as follows:

$$b{+}B_{CT}>\frac{K_{0DT}T}{2}+\frac{3K_{DT}T}{2}+B_{0DT}+3B_{DT} \qquad \text{(AppendixC-12)}$$

Similarly, the stability condition of the m-user haptic virtual surgery simulation system under hybrid control can be derived as follows:

$$\min_i(b_i{+}B_{iCT})>\max_i\{\frac{K_{0iDT}T}{2}{+}B_{0iDT}\}{+}m\max_{i,j\neq i}\{\frac{K_{ijDT}T}{2}{+}B_{ijDT}\} \qquad \text{(AppendixC-13)}$$

$$b+B_{CT}>\frac{K_{DT}T}{2}+B_{DT} \qquad \text{(AppendixC-14)}$$

When $m=1$, the condition presented by (Appendix C-13) becomes Inequality (Appendix C-14) in case of a single user.

C-2 System stability inequality in the active, undelayed scenario

The aforementioned method is applicable for active, delay-free simulation systems. Assuming that the operators are active and the active coefficient is z_{ai} , it can be obtained from (Appendix C-13) that:

$$\min_i(b_i+B_{iCT}-z_{ai})>\max_i\{\tfrac{K_{0iDT}T}{2}+B_{0iDT}\}+m\max_{i,j\neq i}\{\tfrac{K_{ijDT}T}{2}+B_{ijDT}\} \qquad \text{(AppendixC-15)}$$

$$b_d+B_{CT}>\frac{K_{DT}T}{2}+B_{DT} \qquad \text{(AppendixC-16)}$$

The solving process is similar to that in Section C-1, and when $m=1$, this condition becomes Inequality (Appendix C-16) in case of a single user.

C-3 System stability inequality in the passive, delayed scenario

If delays exist in a multi-user haptic virtual surgery simulation system, $t_{di}/T=n_i\neq 0$. It is assumed that n_i is an integer and B_{0iDT} and B_{ijDT} are small. First considering the case of $m=2$, the matrix form of $G^{-1}(z)$ is as follows:

$$G^{-1}(z)=\begin{bmatrix}\left\{\begin{array}{l}(b_1+B_{1CT})\\ +z^{-n_1}\left[\begin{array}{c}\frac{(K_{01DT}+K_{12DT})T}{z-1}\\+\frac{(B_{01DT}+B_{12DT})}{z}\end{array}\right]\end{array}\right\} & -z^{-n_1}\left(\frac{K_{12DT}T}{z-1}+\frac{B_{12DT}}{z}\right)\\ -z^{-n_2}\left(\frac{K_{12DT}T}{z-1}+\frac{B_{12DT}}{z}\right) & \left\{\begin{array}{l}(b_2+B_{2CT})\\ +z^{-n_2}\left[\begin{array}{c}\frac{(K_{02DT}+K_{12DT})T}{z-1}\\+\frac{(B_{02DT}+B_{12DT})}{z}\end{array}\right]\end{array}\right\}\end{bmatrix} \qquad \text{(AppendixC-17)}$$

Since the strict positive realness of $G(z)$ is equivalent to the strict passivity of $G^{-1}(z)$, it is necessary to ensure the strict positive realness of $G^{-1}(z)$. To ensure system stability, it is necessary to verify the positive realness of $G^{-1}(z)$ according to Definition Appendix A-3. Section C-1 has demonstrated that $G^{-1}(z)$ meets Condition 1. The residue matrices obtained from (Appendix C-17) are positive definite matrices, meeting Condition 3. The remaining Condition 2 requires $G^{-1}(e^{j(\omega-\omega_0)T})+G^{-T}(e^{-j(\omega-\omega_0)T})$ to be a positive real matrix. $z=\cos((\omega-\omega_0)T)+j\sin((\omega-\omega_0)T)$ is substituted in Equation (Appendix C-17) and the sum of $G_1^{-1}(e^{j(\omega-\omega_0)T})+G_1^{-T}(e^{-j(\omega-\omega_0)T})$ is checked to determine if it is a positive real matrix. According to Definition Appendix A-2, the following condition is required to be met:

$$t_{d1}=t_{d2} \qquad \text{(AppendixC-18)}$$

$$b_1+B_{1CT}+(B_{01DT}+B_{12DT})\cos((\omega-\omega_0)(t_d-T))\ -\tfrac{(K_{01DT}+K_{12DT})T}{2}\cos((\omega-\omega_0)t_d)-(K_{01DT}+K_{12DT})TS>0$$

(AppendixC-19)

$$\det\left(G^{-1}(e^{j(\omega-\omega_0)T})+G^{-T}(e^{-j(\omega-\omega_0)T})\right)$$
$$=\begin{bmatrix}(b_1+B_{1CT})+B_{01DT}\cos((\omega-\omega_0)(t_d-T))\\ -\frac{K_{01DT}T}{2}\cos((\omega-\omega_0)t_d)-K_{01DT}TS\end{bmatrix}\cdot\begin{bmatrix}(b_2+B_{2CT})+B_{02DT}\cos((\omega-\omega_0)(t_d-T))\\ -\frac{K_{02DT}T}{2}\cos((\omega-\omega_0)t_d)-K_{02DT}TS\end{bmatrix}$$
$$+\begin{bmatrix}B_{12DT}\cos((\omega-\omega_0)(t_d-T))\\ -\frac{K_{12DT}T}{2}\cos((\omega-\omega_0)t_d)-K_{12DT}TS\end{bmatrix}\cdot\begin{bmatrix}(b_1+B_{1CT})+B_{01DT}\cos((\omega-\omega_0)(t_d-T))\\ -\frac{K_{01DT}T}{2}\cos((\omega-\omega_0)t_d)-K_{01DT}TS\end{bmatrix} \quad \text{(AppendixC-20)}$$
$$+\begin{bmatrix}B_{12DT}\cos((\omega-\omega_0)(t_d-T))\\ -\frac{K_{12DT}T}{2}\cos((\omega-\omega_0)t_d)-K_{12DT}TS\end{bmatrix}\cdot\begin{bmatrix}(b_2+B_{2CT})+B_{02DT}\cos((\omega-\omega_0)(t_d-T))\\ -\frac{K_{02DT}T}{2}\cos((\omega-\omega_0)t_d)-K_{02DT}TS\end{bmatrix}>0$$

Here, $S=\frac{\sin((\omega-\omega_0)t_d)\sin((\omega-\omega_0)T)}{(1-\cos((\omega-\omega_0)T))}$. As can be observed from (Appendix C-18), this method demands that the multiple users have different delays. When $\frac{t_d}{T}=n$ is a positive integer, B_{0iDT} and B_{ijDT} are small enough, and $C_{iDT}(z)=K_{DT}+B_{DT}\cdot\frac{z-1}{Tz}$, $i=1,\cdots,m$, the worst case of Inequalities (Appendix C-19) and (Appendix C-20) occurs when S is at its maximum. The maximum value of S can be found by solving $\frac{d}{d(\omega-\omega_0)}S=0$, i.e., by checking the sign of the second derivative of S when $\cos((\omega-\omega_0)T)\to 1$. Therefore, the maximum value of S is as follows:

$$\lim_{\cos((\omega-\omega_0)T)\to 1}\frac{\sin((\omega-\omega_0)t_d)\sin((\omega-\omega_0)T)}{(1-\cos((\omega-\omega_0)T))}=\frac{2t_d}{T} \quad \text{(AppendixC-21)}$$

Therefore,

$$\det\left(G^{-1}(e^{j(\omega-\omega_0)T})+G^{-T}(e^{-j(\omega-\omega_0)T})\right)$$
$$=\left[\;2b+2B_{CT}+2B_{0DT}+B_{12DT}-(K_{01DT}+K_{12DT})T-2(K_{0DT}+K_{12DT})t_d\;\right]^2-[2B_{12DT}-K_{12DT}T-2K_{12DT}t_d]^2>0$$
(AppendixC-22)

Here, $K_{0DT}=\max(K_{01DT},K_{02DT})$, $B_{0DT}=\min(B_{01DT},B_{02DT})$, $b+B_{CT}=\min(b_1+B_{1CT},b_2+B_{2CT})$. Inequality (Appendix C-22) can be simplified as follows:

$$b+B_{CT}+B_{0DT}+2B_{12DT}>\frac{K_{0DT}T}{2}+K_{12DT}T+K_{0DT}t_d+2K_{12DT}t_d \quad \text{(AppendixC-23)}$$

In the same way, the stability condition of the m-user haptic virtual surgery simulation system under hybrid control can be derived as follows:

$$\min_i(b_i+B_{iCT}+B_{0iDT})+m\min_{i,j\neq i}B_{ijDT}>\max_i\{\frac{K_{0iDT}T}{2}+K_{0iDT}t_d\}+m\max_{i,j\neq i}\{\frac{K_{ijDT}T}{2}+K_{ijDT}t_d\} \quad \text{(AppendixC-24)}$$

$$b+B_{CT}+B_{DT}>\frac{K_{DT}T}{2}+K_{DT}t_d \quad \text{(AppendixC-25)}$$

When $m=1$, Condition (Appendix C-24) becomes Inequality (Appendix C-25) in the single-user case.

C-4 System stability inequality in the active, delayed scenario

The analysis method in 3.3 is also applicable to an active, delayed simulation system. Assuming that the operators are active and the active coefficient is z_{ai} , when $\frac{t_d}{T}=n$ is a positive integer, B_{0i} and B_{ij} are small enough, and $C_{iDT}(z)=K_{iDT}+B_{iDT}\cdot\frac{z-1}{Tz}$, $i=1,\cdots,m$, the complete stability condition of the active, delayed haptic virtual surgery simulation system based on FPAA analogue/digital control becomes:

$$\min_i(b_i+B_{iCT}-z_{ai}+B_{0iDT})+m\min_{i,j\neq i}B_{ijDT}>\max_i\{\frac{K_{0iDT}T}{2}+K_{0iDT}t_d\}+m\max_{i,j\neq i}\{\frac{K_{ijDT}T}{2}+K_{ijDT}t_d\} \quad \text{(AppendixC-26)}$$

$$b_d+B_{CT}+B_{DT}>\frac{K_{DT}T}{2}+K_{DT}t_d \quad \text{(AppendixC-27)}$$

The solving process is similar to that described in Section 3.2.1. When $m=1$, the condition becomes Inequality (Appendix C-27) in the single-user scenario.

References

1. H.K. Khalil, *Nonlinear systems third Edition* (Prentice-Hall, 2002).

2. L. Hitz, B.D.O. Anderson, Discrete positive-real functions and their application to system stability, *Proceedings of the Institution of Electrical Engineers*, **116**, 153-155 (1969).
